# Supplementary material for: Checkpoint Proteins Bub1 and Bub3 Delay Anaphase Onset in Response to Low Tension Independent of Microtubule-Kinetochore Detachment
Source: Cell Rep. Author manuscript; Available in PMC 2019 Apr 26. (PMC6485967; doi:10.1016/j.celrep.2019.03.027)
Supplement: 1 [file NIHMS1526693-supplement-1.pdf]

Cell Reports, Volume *27*

Supplemental Information

Checkpoint Proteins Bub1 and Bub3 Delay Anaphase Onset in Response to Low Tension Independent of Microtubule-Kinetochore Detachment

Kathleen G. Proudfoot, Samuel J. Anderson, Sandeep Dave, Angela R. Bunning, Pallavi Sinha Roy, Abesh Bera, and Mohan L. Gupta Jr.

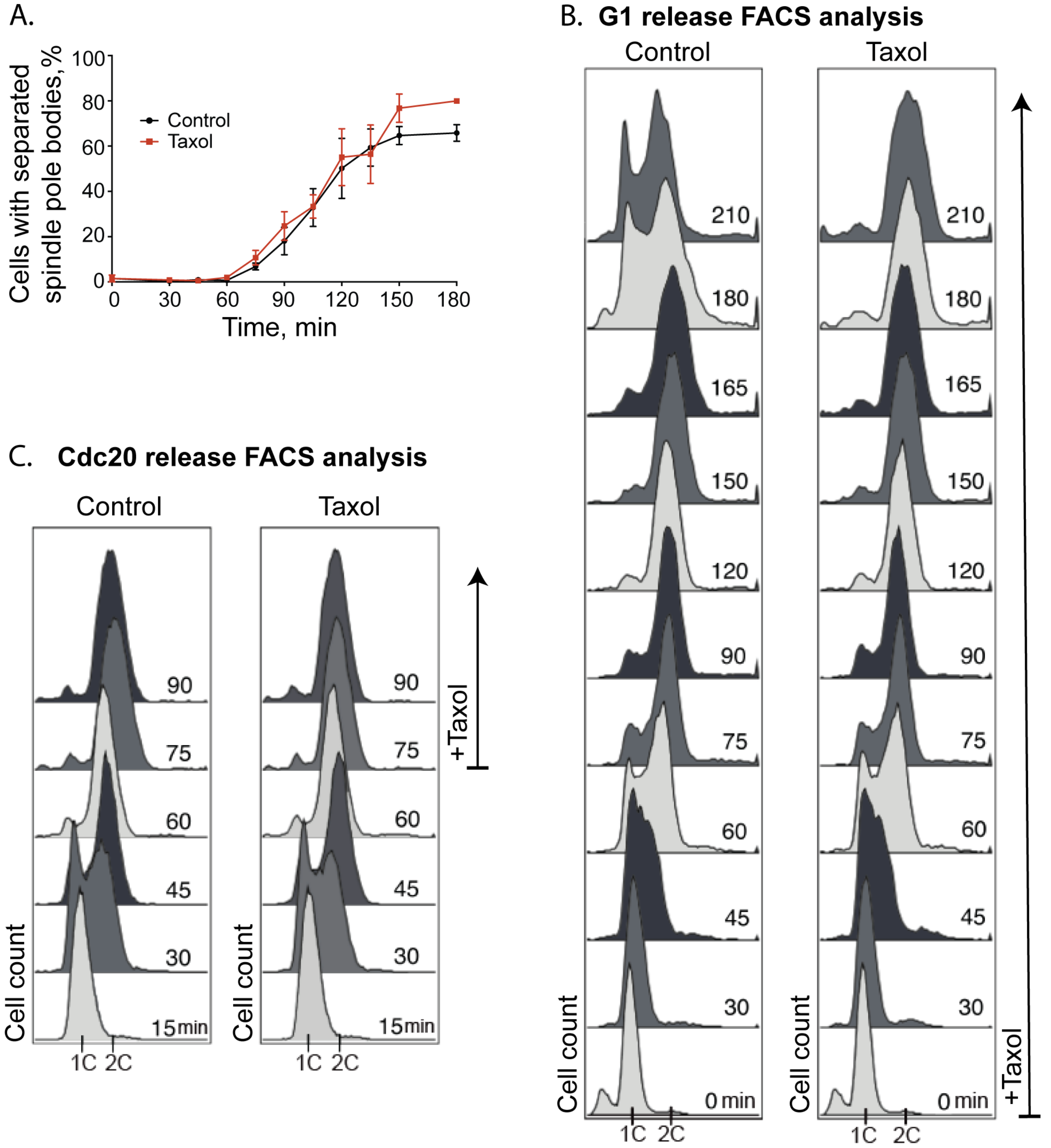

Figure S1. Kinetics of spindle pole body (SPB) separation and DNA replication in the presence and absence of Taxol. Related to Figure 1, Figure 2 and Figure 3. (A) SPB separation during the G1 release assay. Taxol-sensitive cells synchronized in G1 (alpha-factor) were released into the cell cycle and split into sister cultures either with or without 30 μM Taxol. During the ensuing cell cycle SPB separation was monitored by Spc29-mRFP. Experimental scheme of the G1 release assay described in Figure 1E. Plotted values represent the mean ± SEM from 3 experiments, with n = 100-200 cells scored per time point and drug condition for each experiment. (B) DNA replication measured by flow cytometry during the G1 release assay. Taxol was added to the indicated culture at 0 min. DNA replication occurs with similar timing in both cultures and Taxol inhibits cell cycle progression at 180 min. (C) DNA replication measured by flow cytometry during the Cdc20 release assay. Taxol was added to the indicated culture at 75 min. DNA replication occurs with similar timing in both cultures and is essentially complete by 75 min. In (A) and (B) changing colors serve only to create contrast between overlapping histograms.

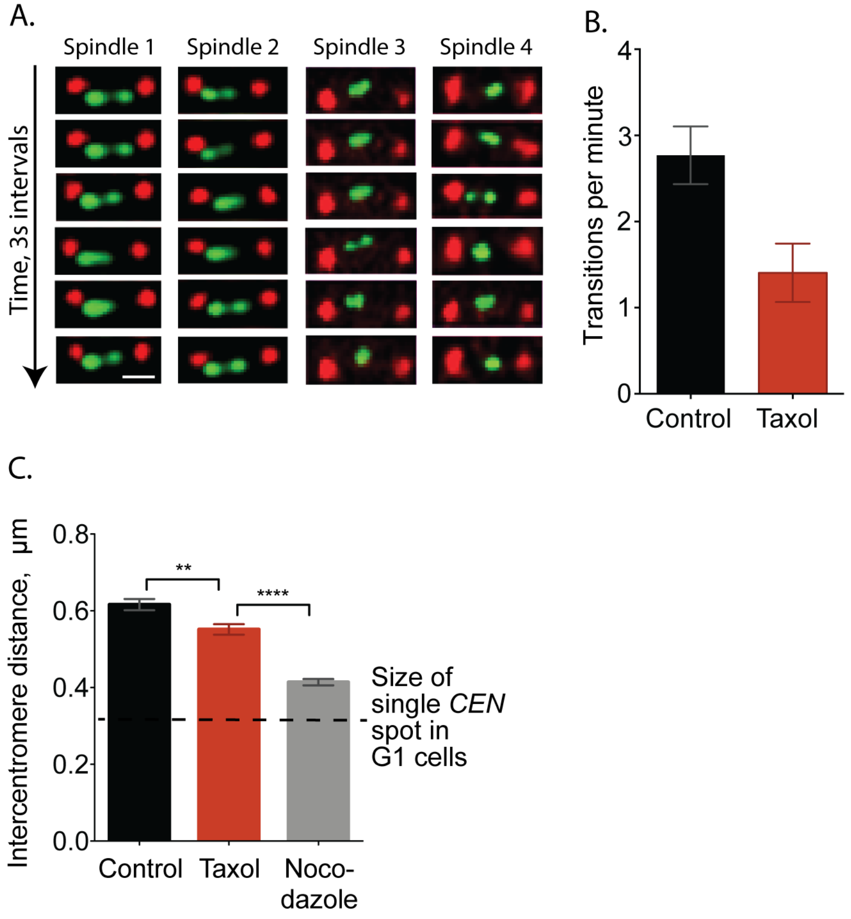

Figure S2. Taxol treatment reduces tension at attached kinetochores. Related to Figure 3. Taxol-sensitive cells were released from G1 synchronization (alpha-factor) into methionine-containing media to hold cells in metaphase with fully formed spindles (Cdc20 depletion). Cultures were split, treated with DMSO ± 30 µM Taxol or 15 µg/ml nocodazole for 15 min, and imaged either live (A-B) or fixed (C). Experimental scheme described in Figure 2A. Sister centromeres were localized by *CEN1*-tetO/tetR-GFP imaging. (A) Representative examples of brief centromere breathing events in metaphase spindles. Spindle poles are red (Spc29-mRFP, outer spots); *CEN1*-GFP is green; time interval between images is 3s; bar = 1 µm. (B) Frequency of transition between either one or two visible *CEN1*-GFP foci. Data represents mean ± SEM observed in individual live cells; n = 31 cells for both; $p = 0.0058$. (C) Distance between the outer edges of *CEN1*-GFP foci in fixed cells (both separated and unseparated). The size of a single, unreplicated *CEN1*-GFP focus in G1 cells is 0.32 ± 0.004 µm (dashed line; n = 170). In nocodazole treated cells, the distance between sister centromeres essentially represents the minimum size of two adjacent *CEN1*-GFP foci in which the majority are tensionless. Mean ± SEM from 4, 4 and 3 experiments for control (n = 178, 156, 145, 169), Taxol (n = 179, 178, 153, 98), and nocodazole (n = 86, 84, 100) treated cells, respectively. $p = 0.0015$ for control versus Taxol, and $p < 0.0001$ for Taxol versus nocodazole treated cells. *p* values were determined by unpaired student's *t*-test (*$p \leq 0.05$, **$p \leq 0.01$, ***$p \leq 0.001$, ****$p \leq 0.0001$).

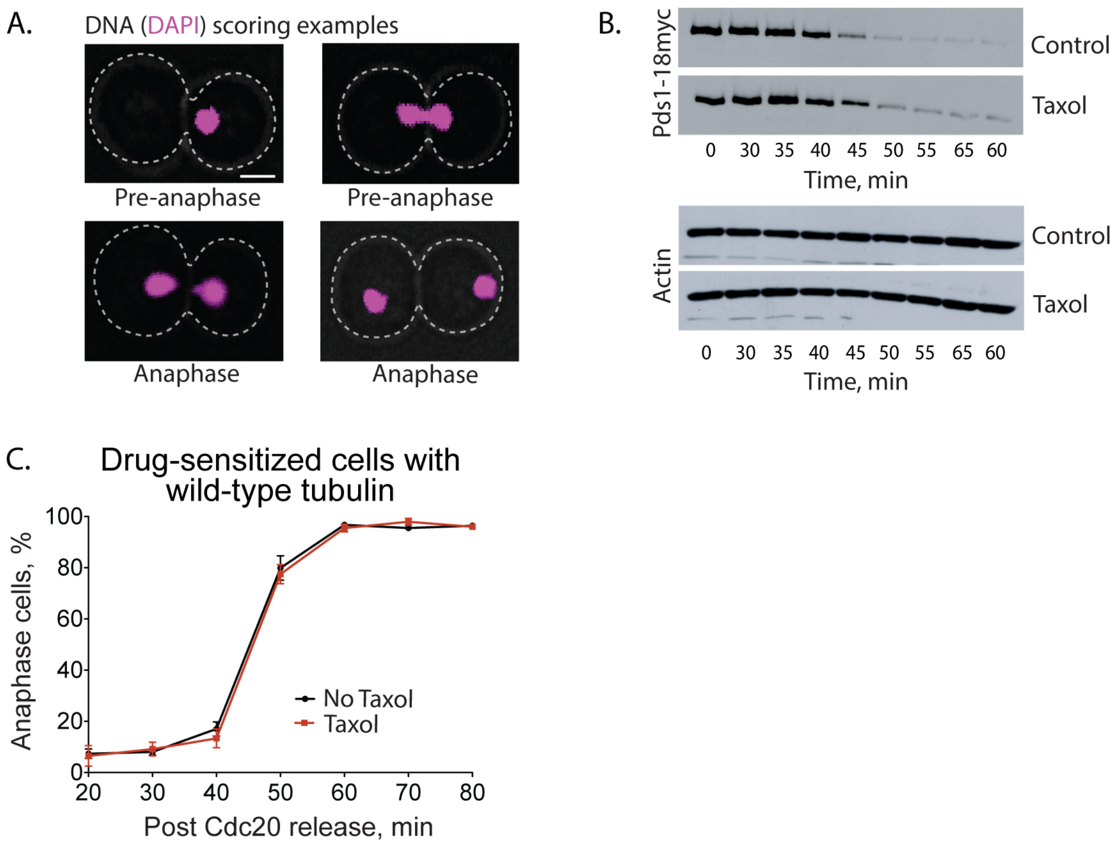

Figure S3. Taxol-induced anaphase delay is dependent on Taxol-tubulin binding. Related to Figure 4. In the Cdc20 release assay, cells synchronized in G1 (alpha-factor) were released into media containing methionine to hold cells in metaphase with fully formed spindles (Cdc20 depletion). Cultures were split, treated with DMSO ± 30 µM Taxol and released from metaphase. Experimental scheme of the Cdc20 release assay described in Figure 4A. (A) Representative images of DAPI-stained cells monitored during the Cdc20 release assay. Bar = 2 µm. (B) Western blot of Pds1-18myc degradation in control and Taxol-treated cells during the Cdc20 release assay. Actin was blotted as a loading control. (C) Timing of anaphase onset in drug-sensitized cells with wild-type yeast tubulin monitored by the Cdc20 release assay (± 30 µM Taxol). Values represent mean ± SEM from 3 experiments, with n = 100-200 cells scored per time point and drug condition for each experiment.

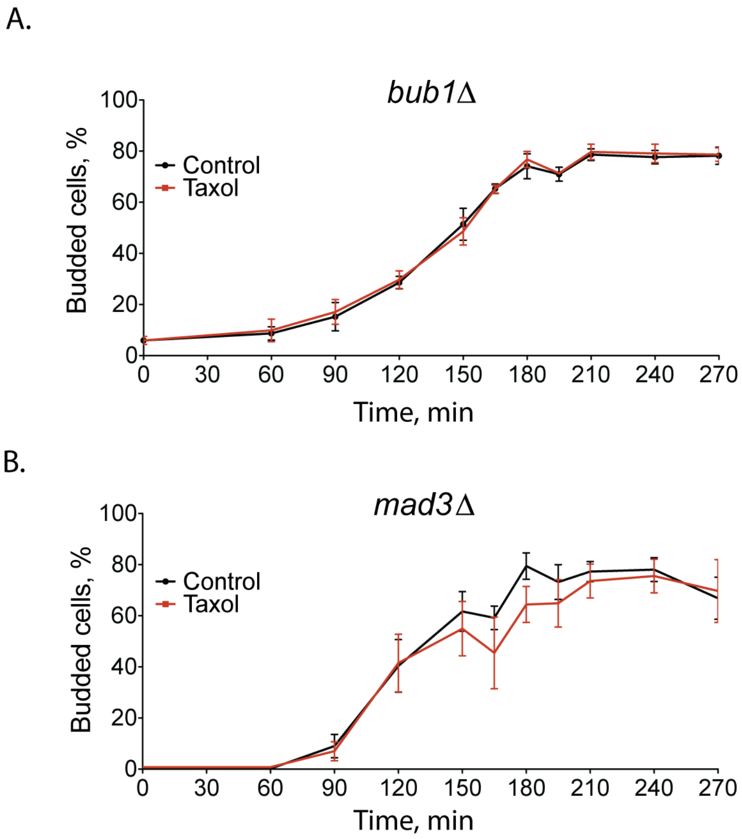

Figure S4. Budding index of cells monitored in the G1 release assay. Related to Figure 5. Taxol-sensitive (A) *bub1*Δ or (B) *mad3*Δ cells synchronized in G1 (alpha-factor) were released into the cell cycle and split into sister cultures either with or without 30 μM Taxol. During the ensuing cell cycle, bud emergence was monitored by cell morphology. Experimental scheme of the G1 release assay described in Figure 1E. Plotted values represent the mean ± SEM from 7 experiments for *bub1*Δ and 6 for *mad3*Δ cells, with n = 100-200 cells scored per time point and drug condition for each experiment.

Table S1. Yeast strains used in this study. Related to STAR Methods.

**Strain**	**Genotype**
MGY50	*MAT***a**, *TUB2, ura3-52*, *his3Δ200*, *leu2Δ1*, *trp1Δ63*
MGY980	*MAT***a**, *tub2-25* (A19K-T23V-G26D-Y270F), *erg6::HIS3, pdr1::hygB, pdr3::NAT, his3Δ200, ura3-52, trp1Δ63, cen1::URA3-CEN3-tetO, pURA3-tetR-YFP-ADHterm-LEU2, SPC29-mRFP-KanR, pMET3-3xHA-CDC20-TRP1*
MGY1293	*MAT***a**, *tub2-25* (A19K-T23V-G26D-Y270F), *erg6::HIS3, pdr1::hygB, pdr3::NAT, his3Δ200, leu2Δ1, ura3-52, trp1Δ63, pMET3-3xHA-CDC20-TRP1*
MGY1315	*MAT***a**, *tub2-25* (A19K-T23V-G26D-Y270F), *erg6::HIS3, pdr1::hygB, pdr3::NAT, his3Δ200, leu2Δ1, ura3-52, trp1Δ63, pMET3-3xHA-CDC20-TRP1, bub1Δ::KanR*
MGY1392	*MAT***a**, *tub2-25* (A19K-T23V-G26D-Y270F), *erg6::HIS3, pdr1::hygB, pdr3::NAT, his3Δ200, leu2Δ1, ura3-52, trp1Δ63, pMET3-3xHA-CDC20-TRP1, PDS1-18myc-LEU2*
MGY1399	*MAT***a**, *tub2-25* (A19K-T23V-G26D-Y270F), *erg6::TRP1, pdr1::hygB, pdr3::NAT, his3Δ200, leu2Δ1, trp1Δ63, GFP-TUB1-URA3*
MGY1428	*MAT***a**, *TUB2, erg6::HIS3, pdr1::hygB, pdr3::NAT, his3Δ200, leu2Δ1, ura3-52, trp1Δ63, pMET3-3xHA-CDC20-TRP1*
MGY1508	*MAT***a**, *tub2-25* (A19K-T23V-G26D-Y270F), *erg6::HIS3, pdr1::hygB, pdr3::NAT, his3Δ200, leu2Δ1, ura3-52, trp1Δ63, pMET3-3xHA-CDC20-TRP1, mad3Δ::KanR*
MGY1693	*MAT***a**, *tub2-25* (A19K-T23V-G26D-Y270F), *erg6::HIS3, pdr1::hygB, pdr3::NAT, his3Δ200, leu2Δ1, ura3-52, trp1Δ63, pMET3-3xHA-CDC20-TRP1, mad1Δ::KanR*
MGY1739	*MAT***a**, *tub2-25* (A19K-T23V-G26D-Y270F), *erg6::HIS3, pdr1::hygB, pdr3::NAT, his3Δ200, leu2Δ1, ura3-52, trp1Δ63, pMET3-3xHA-CDC20-TRP1, bub3Δ::KanR*
MGY1749	*MAT***a**, *tub2-25* (A19K-T23V-G26D-Y270F), *erg6::HIS3, pdr1::hygB, pdr3::NAT, his3Δ200, leu2Δ1, ura3-52, trp1Δ63, cen1::URA3-CEN3-tetO, 5'NLS-tetR-GFP-ADHterm-LEU2, SPC29-mRFP-KanR, pMET3-3xHA-CDC20-TRP1*
MGY1830	*MAT***a**, *tub2-25* (A19K-T23V-G26D-Y270F), *erg6::HIS3, pdr1::hygB, pdr3::NAT, his3Δ200, leu2Δ1, ura3-52, trp1Δ63, pMET3-3xHA-CDC20-TRP1, mad2Δ::KanR*
MGY1872	*MAT***a**, *TUB2, erg6::TRP1, pdr1::hygB, pdr3::NAT, his3Δ200, leu2Δ1, ura3-52, trp1Δ63*
MGY2103	*MAT***a**, *tub2-25* (A19K-T23V-G26D-Y270F), *erg6::TRP1, pdr1::hygB, pdr3::NAT, his3Δ200, leu2Δ1, ura3-52, trp1Δ63, bub3Δ::KanR*
MGY2127	*MAT***a**, *tub2-25* (A19K-T23V-G26D-Y270F), *erg6::HIS3, pdr1::hygB, pdr3::NAT, his3Δ200, leu2Δ1, ura3-52, trp1Δ63, cen1::URA3-CEN3-tetO, 5'NLS-tetR-GFP-ADHterm-LEU2, pMET3-3xHA-CDC20-TRP1, mad3Δ::KanR*
MGY2128	*MAT***a**, *tub2-25* (A19K-T23V-G26D-Y270F), *erg6::TRP1, pdr1::hygB, pdr3::NAT, his3Δ200, leu2Δ1, ura3-52, trp1Δ63, bub1Δ::KanR*
MGY2133	*MAT***a**, *tub2-25* (A19K-T23V-G26D-Y270F), *erg6::TRP1, pdr1::hygB, pdr3::NAT, his3Δ200, leu2Δ1, ura3-52, trp1Δ63, mad1Δ::KanR*
MGY2134	*MAT***a**, *tub2-25* (A19K-T23V-G26D-Y270F), *erg6::TRP1, pdr1::hygB, pdr3::NAT, his3Δ200, leu2Δ1, ura3-52, trp1Δ63, mad2Δ::KanR*
MGY2136	*MAT***a**, *tub2-25* (A19K-T23V-G26D-Y270F), *erg6::TRP1, pdr1::hygB, pdr3::NAT, his3Δ200, leu2Δ1, ura3-52, trp1Δ63, mad3Δ::KanR*
MGY2139	*MAT***a**, *tub2-25* (A19K-T23V-G26D-Y270F), *erg6::TRP1, pdr1::hygB, pdr3::NAT, his3Δ200, leu2Δ1, ura3-52, trp1Δ63, mad3Δ::KanR, bub1Δ::KanR*
MGY2150	*MAT***a**, *tub2-25* (A19K-T23V-G26D-Y270F), *erg6::TRP1, pdr1::hygB, pdr3::NAT, his3Δ200, leu2Δ1, ura3-52, trp1Δ63*
MGY2226	*MAT***a**, *tub2-25* (A19K-T23V-G26D-Y270F), *erg6::HIS3, pdr1::hygB, pdr3::NAT, his3Δ200, leu2Δ1, ura3-52, trp1Δ63, pMET3-3xHA-CDC20-TRP1, GFP-TUB1-URA3*
MGY2227	*MAT***a**, *tub2-25* (A19K-T23V-G26D-Y270F), *erg6::HIS3, pdr1::hygB, pdr3::NAT, his3Δ200, leu2Δ1, ura3-52, trp1Δ63, pMET3-3xHA-CDC20-TRP1, bub1Δ::KanR, GFP-TUB1-URA3*
MGY2228	*MAT***a**, *tub2-25* (A19K-T23V-G26D-Y270F), *erg6::HIS3, pdr1::hygB, pdr3::NAT, his3Δ200, leu2Δ1, ura3-52, trp1Δ63, pMET3-3xHA-CDC20-TRP1, mad2Δ::KanR, GFP-TUB1-URA3*

All strains except MGY50 (control S288C strain) were created in this study.
