# Supplementary material for: Checkpoint Proteins Bub1 and Bub3 Delay Anaphase Onset in Response to Low Tension Independent of Microtubule-Kinetochore Detachment
Source: Cell Rep. Author manuscript; Available in PMC 2019 Apr 26. (PMC6485967; doi:10.1016/j.celrep.2019.03.027)
Supplement: 2 [file NIHMS1526693-supplement-2.pdf]

Cell Reports

Article

Checkpoint Proteins Bub1 and Bub3 Delay Anaphase Onset in Response to Low Tension Independent of Microtubule-Kinetochore Detachment

Graphical Abstract

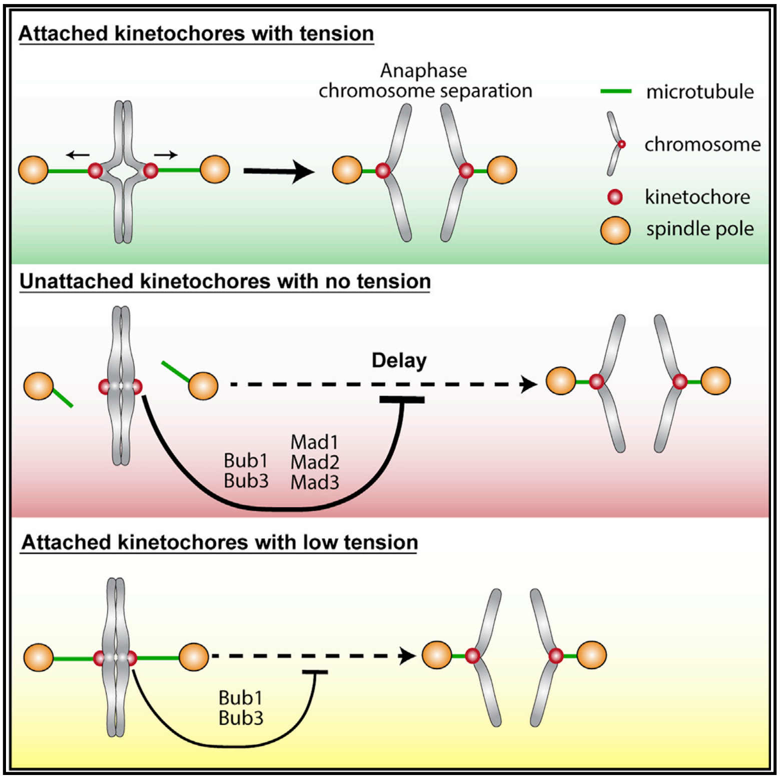

Highlights

- Taxol-sensitive yeast model allows separation of kinetochore tension and attachment
- Low tension at attached kinetochores generates a delay in anaphase onset
- The tension-specific delay mechanism requires the SAC proteins Bub1 and Bub3
- The tension mechanism does not require the core SAC proteins Mad1, Mad2, and Mad3

Authors

Kathleen G. Proudfoot,
Samuel J. Anderson, Sandeep Dave,
Angela R. Bunning, Pallavi Sinha Roy,
Abesh Bera, Mohan L. Gupta, Jr.

Correspondence

mgupta@iastate.edu

In Brief

Kinetochore attachment and tension are critical for proper chromosome segregation, but isolating the contribution of either stimulus has been challenging. Using a Taxol-sensitive yeast model, Proudfoot et al. show that reducing tension specifically produces a delay in mitotic progression that is temporally and mechanistically distinct from that produced by unattached kinetochores.

Proudfoot et al., 2019, Cell Reports *27*, 416–428
April 9, 2019 © 2019 The Author(s).
https://doi.org/10.1016/j.celrep.2019.03.027

CellPress

OPEN ACCESS
CellPress

Cell Reports
Article

Checkpoint Proteins Bub1 and Bub3 Delay Anaphase Onset in Response to Low Tension Independent of Microtubule-Kinetochore Detachment

Kathleen G. Proudfoot,[1,2] Samuel J. Anderson,[1,3] Sandeep Dave,[1,3] Angela R. Bunning,[1] Pallavi Sinha Roy,[1] Abesh Bera,[1] and Mohan L. Gupta, Jr.[1,4,*]
[1]Genetics, Development, and Cell Biology, Iowa State University, Ames, IA 50011, USA
[2]Molecular Genetics and Cell Biology, University of Chicago, Chicago, IL 60637, USA
[3]These authors contributed equally
[4]Lead Contact
*Correspondence: mgupta@iastate.edu
https://doi.org/10.1016/j.celrep.2019.03.027

SUMMARY

The spindle assembly checkpoint (SAC) delays anaphase onset until sister chromosomes are bound to microtubules from opposite spindle poles. Only then can dynamic microtubules produce tension across sister kinetochores. The interdependence of kinetochore attachment and tension has proved challenging to understanding SAC mechanisms. Whether the SAC responds simply to kinetochore attachment or to tension status remains obscure. Unlike higher eukaryotes, budding yeast kinetochores bind only one microtubule, simplifying the relation between attachment and tension. We developed a Taxol-sensitive yeast model to reduce tension in fully assembled spindles. Our results show that low tension on bipolar-attached kinetochores delays anaphase onset, independent of detachment. The delay is transient relative to that imposed by unattached kinetochores. Furthermore, it is mediated by Bub1 and Bub3, but not Mad1, Mad2, and Mad3 (BubR1). Our results demonstrate that reduced tension delays anaphase onset via a signal that is temporally and mechanistically distinct from that produced by unattached kinetochores.

INTRODUCTION

Accurate chromosome segregation is critical to cell division. Missegregation leads to aneuploidy, birth defects, and tumor progression (Gordon et al., 2012; Siegel and Amon, 2012). In eukaryotes, faithful segregation requires that the kinetochores of sister chromosomes attach to microtubules emanating from opposite spindle poles. Only in this bipolar configuration can dynamic microtubules generate tension across the sister kinetochores (Figures 1A and 1B). To ensure proper segregation, a surveillance mechanism, called the spindle assembly checkpoint (SAC), signals to delay anaphase onset under conditions in which either attachment or tension is lacking (Figure 1B). It has been a long-standing challenge to understand how the tension status contributes to kinetochore-based signaling and/or SAC activation. It is widely accepted that unattached kinetochores activate the SAC (London and Biggins, 2014a). Studies addressing the role of tension have produced contradictory evidence (Biggins and Murray, 2001; Etemad et al., 2015; King et al., 2007; Li and Nicklas, 1995; Magidson et al., 2016; Maresca and Salmon, 2009; Nicklas et al., 1995; O'Connell et al., 2008; Pinsky et al., 2006; Rieder et al., 1994, 1995; Shannon et al., 2002; Skoufias et al., 2001; Stern and Murray, 2001; Suzuki et al., 2016; Tauchman et al., 2015; Uchida et al., 2009; Wan et al., 2009; Waters et al., 1998), and a consensus has not been obtained (Khodjakov and Pines, 2010; Krenn and Musacchio, 2015; Maresca and Salmon, 2010; Murray, 2011; Nezi and Musacchio, 2009). Experiments using micro-manipulation in praying mantis spermatocytes (Li and Nicklas, 1995) or unpaired chromosomes in yeast (Shonn et al., 2000; Stern and Murray, 2001) provide compelling evidence that reduced tension results in SAC activation. However, the interpretation of these experiments has been confounded by the error-correction mechanism in which tensionless microtubule-kinetochore attachments are selectively destabilized by the activity of Aurora B kinase (Biggins and Murray, 2001; Krenn and Musacchio, 2015; Pinsky et al., 2006; Tanaka et al., 2002). This central caveat has generally prevented the exclusion of unattached kinetochores as a SAC signal under conditions of reduced tension. Notably, unattached kinetochores themselves are not under tension. Thus, whether a lack of tension contributes directly to SAC signaling mechanism(s) and/or a delay in anaphase onset, independent of inducing kinetochore detachment, remains obscure (Figure 1C).

The microtubule stabilizer paclitaxel (Taxol) reduces tension at kinetochore attachments and activates the SAC in higher eukaryotes (Maresca and Salmon, 2009; Waters et al., 1998). Kinetochores in these cells typically bind dozens of microtubules, and decreased tension is correlated with reduced binding occupancy (King and Nicklas, 2000; Nicklas and Ward, 1994; Nicklas et al., 2001). Although 1-h Taxol treatment in mammalian PtK1 cells does not reduce the average, it increases the variation in the number of microtubules bound to each kinetochore (McEwen et al., 1997), and it is not known to what extent the unoccupied binding sites resulting from altered microtubule occupancy may be

This is an open access article under the CC BY license (http://creativecommons.org/licenses/by/4.0/).

OPEN
ACCESS
CellPress

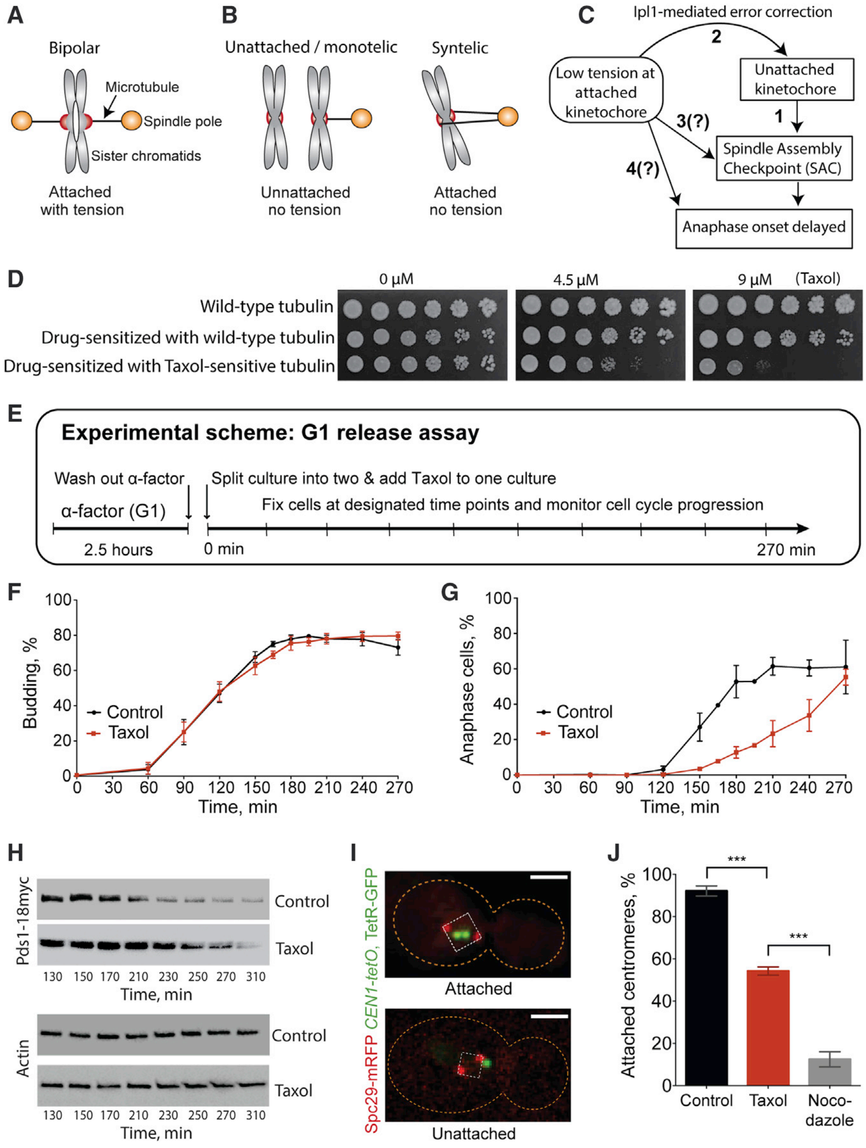

Figure 1. Taxol Treatment during Spindle Assembly Delays Anaphase Onset with Unattached and Low-Tension Kinetochores Present

(A) When sister kinetochores attach to microtubules from opposite spindle poles, dynamic microtubules can generate tension across the kinetochores.

(B) Improper attachments: if one or both kinetochores are unattached (left) or both are attached to microtubules emanating from the same pole (right), microtubules cannot generate tension across sister kinetochores.

(C) Pathways for kinetochore-related signaling. (1) Unattached kinetochores activate the SAC. (2) Kinetochore attachments with insufficient tension are destabilized via the Ipl1 (Aurora B)-mediated error-correction pathway. In addition, attached kinetochores with insufficient tension may (3) directly activate the SAC or (4) delay anaphase onset without activating the canonical SAC.

(D) Serial dilutions of control cells with wild-type tubulin, drug-sensitized cells with wild-type tubulin, and drug-sensitized cells with Taxol-sensitive tubulin (Taxol-sensitive cells) were spotted on plates with the indicated Taxol concentration.

(E) Experimental scheme of the G1 release assay.

(F and G) Timing of bud emergence (F) and anaphase onset (G) in Taxol-sensitive cells monitored by the G1 release assay (±30 μM Taxol). Means ± SEMs of 5 experiments, with n = 100–200 cells scored per time point and drug condition for each experiment.

(H) Western blot of Pds1-18myc and actin (loading control) during the G1 release assay, as in (G).

(I) Fluorescence micrographs of metaphase cells. The cell outline is indicated by the orange dashed line; spindle poles are red (in box corners); a single centromere pair is green (*CEN1*); a box (white, dashed line) was generated by right angles emanating from each pole. Centromeres were considered attached if both *CEN1* spots were inside and detached if spots were outside this box. Bar, 2 μm.

(J) Percentage of cells with attached centromeres when Taxol or nocodazole is present during spindle assembly. Log phase cultures of Taxol-sensitive cells were incubated for 2 h in the absence (control) or presence of Taxol or nocodazole. Cells were fixed and centromere attachment (via associated kinetochores) was scored as in (I). Means ± SEMs from 3 experiments; n = 114, 81, and 108 (control); 107, 118, and 106 (Taxol); and 130, 113, and 125 (nocodazole); p = 0.0003 and 0.0005 for Taxol versus control and nocodazole-treated cells, respectively.

See also Figure S1.

recognized as an unattached kinetochore. Evidence also indicates that prolonged mitotic arrest in Taxol is associated with unattached kinetochores (Magidson et al., 2016; Waters et al., 1998). Thus, unoccupied binding sites, rather than a lack of tension, cannot be excluded as a SAC signal. Unlike higher eukaryotes, the budding yeast *Saccharomyces cerevisiae* kinetochore binds just one microtubule (Winey et al., 1995). This simplifies the relation between tension and attachment because the budding yeast kinetochore is either fully attached or unattached; there is no reduced microtubule occupancy. Microtubule stabilizers, however, have not been used to study the SAC in yeast because Taxol does not stabilize wild-type yeast microtubules (Barnes et al., 1992; Bode et al., 2002).

To address the role of microtubule-generated tension in the SAC, we used a Taxol-sensitive budding yeast model harboring an engineered β-tubulin that allows Taxol stabilization of yeast microtubules (Foland et al., 2005; Gupta et al., 2003). Unlike previous approaches that disrupt bipolar spindle organization (Piatti et al., 1995; Stern and Murray, 2001; Uhlmann et al., 2000), our approach allows the tension status to be modulated in preformed spindles and the timing of anaphase onset to be directly monitored at the single-cell level. We find that reduced tension on bipolar-attached yeast kinetochores delays anaphase onset, independent of kinetochore detachment. This delay is shorter than that imposed by unattached kinetochores. It requires the SAC proteins Bub1 and Bub3, but persists in the absence of

Mad1, Mad2, or Mad3 (BubR1), which are required for the response to unattached kinetochores. Thus, the tension-mediated delay is temporally and mechanistically distinct from the canonical SAC response to unattached kinetochores.

RESULTS AND DISCUSSION

Taxol does not bind to wild-type yeast tubulin (Barnes et al., 1992; Bode et al., 2002). By modifying five amino acids, we previously created an active binding site in yeast β-tubulin that allows Taxol to robustly stabilize yeast microtubules *in vitro* (Gupta et al., 2003). The growth of yeast harboring this tubulin is not inhibited because cells efficiently export the drug, but placing this tubulin in a strain deficient for the pleiotropic drug response (ABC transmembrane transporters) renders yeast cells sensitive to Taxol (Foland et al., 2005). Although this modified tubulin does not significantly perturb microtubule dynamics in the absence of Taxol (Entwistle et al., 2012), treatment with the drug causes resistance to cold-induced depolymerization and delays mitotic progression (Foland et al., 2005). As in higher eukaryotes, Taxol stabilizes microtubules and induces mitotic delay in Taxol-sensitive budding yeast.

To examine the effect of microtubule stabilization on mitotic progression, we sensitized the commonly used S288C background to Taxol. Similar to previous results, Taxol does not inhibit the growth of "drug-sensitized" yeast (*pdr1Δ pdr3Δ erg6Δ*) that displays increased sensitivity to exogenous compounds (Figure 1D). The addition of Taxol-sensitive tubulin, however, results in Taxol sensitivity (Figure 1D).

Taxol Treatment during Spindle Assembly Delays Anaphase Onset with Unattached and Low-Tension Kinetochores Present

In higher eukaryotes, Taxol inhibits spindle assembly and produces a SAC-dependent delay in anaphase onset (Fuchs and Johnson, 1978; Jordan et al., 1993; Schiff and Horwitz, 1980; Waters et al., 1998). To monitor the timing of anaphase onset in Taxol-treated yeast, we used a G1 release assay (Figure 1E). We released G1-synchronized cells into the cell cycle, split them into sister cultures either with or without Taxol, and monitored cell-cycle progression. We found Taxol to be more effective on agar plates than in liquid media. Thus, experiments in liquid were conducted using 30 μM Taxol. Previous approaches to reduce tension at kinetochores used unreplicated chromosomes or diminished cohesion, which severely disrupts spindle organization and results in premature spindle elongation; this obscures the direct observation of anaphase onset (Piatti et al., 1995; Stern and Murray, 2001; Uhlmann et al., 2000). Microtubule destabilizers such as nocodazole prevent spindle assembly and thus similarly prohibit the visualization of anaphase onset. Such approaches typically rely instead on the analysis of biochemical markers (e.g., Pds1 [Securin]) in bulk culture to detect the transition into anaphase. In contrast, 30 μM Taxol treatment delayed but did not prevent spindle assembly, which allowed anaphase elongation and chromosome separation to be directly monitored in individual cells. The budding index reveals that Taxol does not affect the timing of cell-cycle initiation (Figure 1F). However, anaphase onset was significantly delayed by Taxol treatment (Figure 1G). Pds1 degradation was similarly delayed, confirming that chromosome segregation is an accurate marker of anaphase onset in individual cells (Figure 1H). Spindle pole body (SPB) separation occurred with similar kinetics under both conditions, indicating that the delay does not result from slowed SPB duplication or initiation of spindle assembly (Figure S1A). DNA replication was also completed simultaneously in control and Taxol-treated cultures (Figure S1B). Thus, Taxol treatment delays anaphase onset by approximately 1 h, and, similar to higher eukaryotes, this delay results from slowed spindle assembly.

To examine the effects of Taxol on spindle assembly, we labeled spindle poles (Spc29-monomeric red fluorescent protein [mRFP]) and monitored sister centromeres using a tetO/tetR-GFP array integrated 228 bp from *CEN1* (Hsu et al., 2003), which is sufficiently positioned to reflect microtubule-dependent sister separation (Pearson et al., 2001). Centromere alignment on the spindle axis between the poles is indicative of kinetochore attachment (Goshima and Yanagida, 2000; He et al., 2000; Pearson et al., 2001; Tanaka et al., 2002). Imaging of attached centromeres in live cells revealed that they typically lie up to ~300 nm off the pole-to-pole axis (Haase et al., 2012). Therefore, we scored centromeres and their associated kinetochores as attached if they were within a square created by extending lines from each pole toward the central spindle (Figure 1I). In cycling cells, Taxol reduced centromere attachment nearly 50% in pre-anaphase cells with separated SPBs (Figure 1J). As a control, treatment with the microtubule destabilizer nocodazole reduced attachment 90% (Figure 1J). Microtubules cannot generate tension on unattached kinetochores. Thus, Taxol treatment during spindle assembly results in kinetochores that are both unattached and under low tension, and either condition could contribute to the observed delay in anaphase onset.

Taxol Treatment of Preformed Spindles Does Not Induce Kinetochore Detachment

We sought to exploit the tractability of yeast and the fact that budding yeast kinetochores bind only one microtubule to control the tension status of attached kinetochores. Cdc20 activates the anaphase-promoting complex (APC) to drive cells from metaphase to anaphase. Cells depleted for Cdc20 are unable to enter anaphase, even though spindle assembly is complete and the SAC is satisfied (Lim et al., 1998). We released G1-synchronized cells into methionine-containing media to repress *CDC20* expression (*pMET3-CDC20*). After 60 min, the vast majority of cells displayed metaphase spindles with *CEN1* properly attached. To ensure complete spindle assembly in the absence of microtubule poisons, we allowed an additional 15 min. Flow cytometry analysis confirmed that DNA replication was complete within this time (Figure S1C). We then split the culture and treated half with Taxol (Figure 2A). Spindle morphology remains comparable in both cultures (Figure 2B). However, observation of individual astral microtubules showed that the Taxol treatment suppresses microtubule dynamics (Figures 2C and 2D). Specifically, the catastrophe frequency and depolymerization rate are decreased by ~70%, the time spent in the attenuated state is increased from 20%

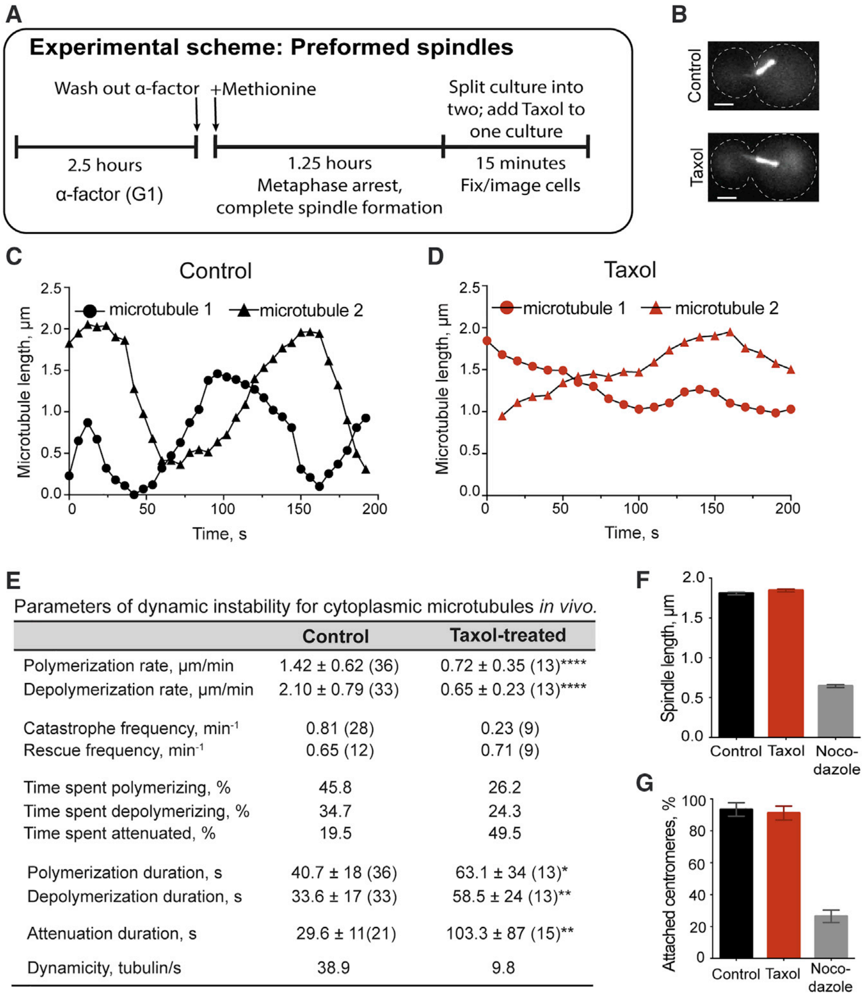

	Control	Taxol-treated
Polymerization rate, μm/min	1.42 ± 0.62 (36)	0.72 ± 0.35 (13)****
Depolymerization rate, μm/min	2.10 ± 0.79 (33)	0.65 ± 0.23 (13)****
Catastrophe frequency, min^{-1}	0.81 (28)	0.23 (9)
Rescue frequency, min^{-1}	0.65 (12)	0.71 (9)
Time spent polymerizing, %	45.8	26.2
Time spent depolymerizing, %	34.7	24.3
Time spent attenuated, %	19.5	49.5
Polymerization duration, s	40.7 ± 18 (36)	63.1 ± 34 (13)*
Depolymerization duration, s	33.6 ± 17 (33)	58.5 ± 24 (13)**
Attenuation duration, s	29.6 ± 11(21)	103.3 ± 87 (15)**
Dynamicity, tubulin/s	38.9	9.8

Figure 2. Taxol Treatment of Preformed Spindles Does Not Induce Kinetochore Detachment

(A) Schematic of Taxol treatment of preformed metaphase spindles. G1-synchronized cells were released into media containing methionine to hold cells in metaphase with fully formed spindles (Cdc20 depletion). Cultures were split, treated with DMSO ± 30 μM Taxol or 15 μg/mL nocodazole for 15 min and imaged live (B–E) or fixed (F and G).

(B) Metaphase spindles in control and Taxol-treated cells viewed by GFP-labeled microtubules (GFP-Tub1). Bars, 2 μm.

(C and D) Representative lifetime history plots for astral microtubules in metaphase cells in the (C) absence or (D) presence of Taxol.

(E) Microtubule dynamic instability parameters in control and Taxol-treated metaphase cells. Means ± SDs; sample number in parentheses; 3,196 (control) and 3,130 (Taxol-treated) s of total microtubule lifetime were analyzed; *$p \leq 0.05$, **$p \leq 0.01$, ****$p \leq 0.0001$.

(F) Spindle length measured from outer edges of Spc29-mRFP marked spindle pole bodies. Means ± SEMs; n = 469 (control), 429 (Taxol), and 258 (nocodazole).

(G) Percentage of cells with attached centromeres when Taxol or nocodazole is added to preformed spindles. The attachment is measured as in Figure 1I. Means ± SEMs from 3 experiments; n = 111, 110, and 155 (control); 148, 100, and 178 (Taxol); and 100, 58, and 100 (nocodazole).

See also Figure S1.

to 50%, and overall dynamicity is reduced 75% (Figure 2E). Despite these changes, spindle length is unchanged in the metaphase-arrested cells, indicating that Taxol treatment does not significantly disrupt the architecture of preformed spindles (Figure 2F). Notably, centromere attachment to preformed spindles remains similar in control and Taxol-treated cells (Figure 2G). In contrast, nocodazole treatment of preformed spindles reduces their length by 70% and dramatically induces centromere detachment (Figures 2F and 2G). Thus, under these conditions, Taxol treatment does not cause significant kinetochore detachment from preformed yeast spindles.

Taxol Treatment Reduces Tension at Attached Kinetochores in Preformed Spindles

We next examined the tension status of kinetochores in preformed spindles treated with Taxol. In diverse organisms, the action of dynamic microtubules increases the distance between bipolar-attached sister centromeres, which generates tension across the centromeres and kinetochores (Goshima and Yanagida, 2000; Kelling et al., 2003; Maresca and Salmon, 2009; Uchida et al., 2009; Wan et al., 2009; Waters et al., 1998). Suppressing microtubule dynamics decreases this tension. In budding yeast, bipolar-attached centromeres oscillate between states of increased separation, in which centromere-associated tetO/tetR-GFP signals can be resolved as two distinct foci, and decreased separation, when they are close enough that their diffraction-limited spots overlap (Figure 3A) (He et al., 2000; Tanaka et al., 2000). Kymographs from time-lapse imaging of control spindles reveal centromere oscillation between separated and unseparated GFP foci (Figure 3B). Consistent with suppressed microtubule dynamics, centromeres appear to oscillate less in Taxol-treated spindles (Figure 3B). When observed by time-lapse imaging, Taxol reduces the time that centromeres spend in the separated state (Figure 3C). The frequency of transition between the two states is also reduced (Figures S2A and S2B). Similarly, in fixed cells Taxol reduces the percentage of cells with separated centromere-associated foci by 36% (Figure 3D), along with the average distance between sister centromeres (Figure S2C). As a control, nocodazole, which completely eliminates tension by inducing kinetochore detachment, reduces this distance significantly more than Taxol and closely approximates the minimum size for two adjacent *CEN1*-GFP foci (Figure S2C). Finally, in the fraction of cells displaying separated centromeres, we measured the distance between the centroids of the two foci. Not only

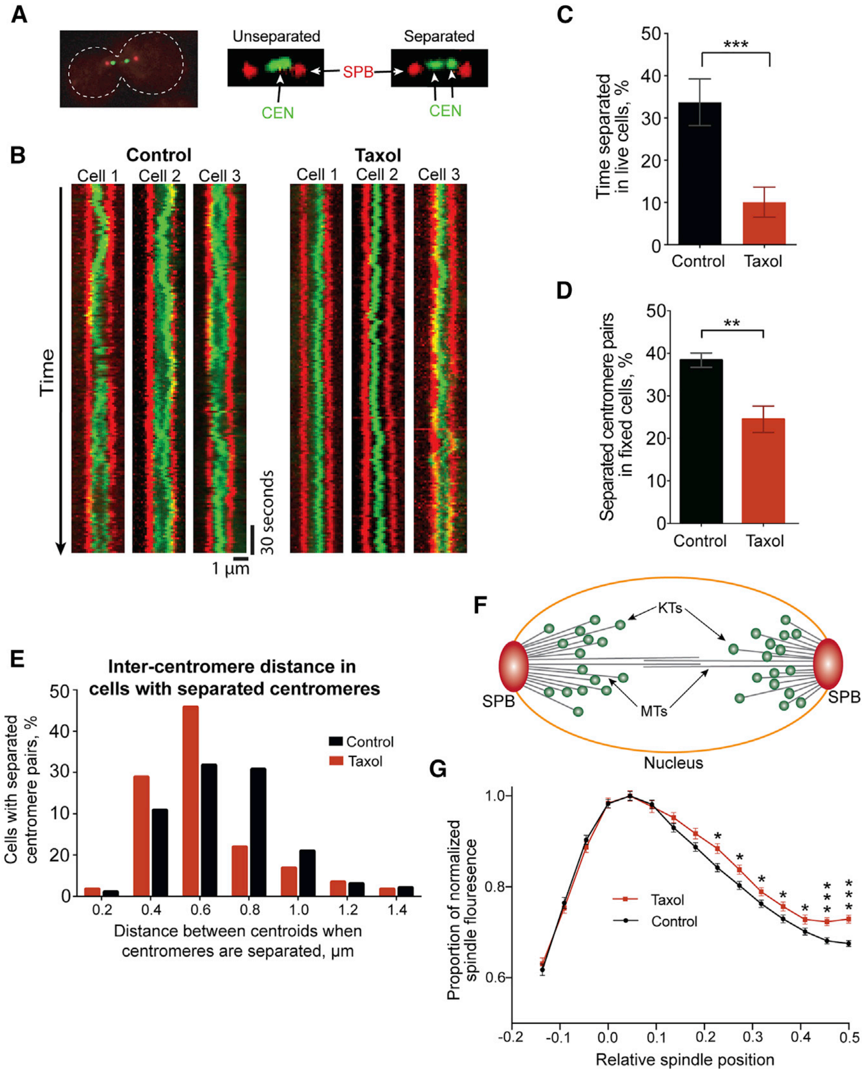

Figure 3. Taxol Treatment Reduces Tension at Attached Kinetochores in Preformed Spindles

Preformed spindles were prepared as in Figure 2A.

(A) Example images of separated and unseparated *CEN1* pairs.

(B) Kymographs of metaphase spindles in control and Taxol-treated (30 μM) cells. Spindle pole marked with Spc29-mRFP (red); *CEN1* marked with GFP (green).

(C) Percentage of time that sister *CEN1*-GFP foci are separated on metaphase spindles in live cells. Means ± SEMs of values observed in individual cells; n = 31 for both; p = 0.0007.

(D) Percentage of fixed metaphase cells with separated sister *CEN1*-GFP foci. Means ± SEMs from 3 experiments; n = 111, 110, and 155 (control); 148, 100, and 178 (Taxol); p = 0.008.

(E) Distance between *CEN1*-GFP centroids measured only in cells with separated *CEN1*-GFP foci. Means ± SEMs = 0.70 ± 0.02 and 0.63 ± 0.03 for control and Taxol treated, respectively; p = 0.024; n = 101 (control) and 59 (Taxol).

(F) Schematic depicting SPBs, microtubules (MTs), and kinetochores (KTs) in yeast metaphase spindle.

(G) GFP-labeled microtubule fluorescence measured by spindle position. The outer edge of each spindle is designated position 0.0 and 0.5 is the spindle equator. Means ± SEMs from 82 (control) and 78 (Taxol) spindles. For indicated positions, *p ≤ 0.05, ***p ≤ 0.001.

See also Figures S1 and S2.

are there fewer cells with separated foci during Taxol treatment, but they are also not pulled as far apart as those in control cells (Figure 3E). These observations are consistent with the mechanism of action of Taxol in inhibiting microtubule depolymerization. Low doses of microtubule destabilizers such as benomyl can stabilize microtubules but also promote depolymerization (Gupta et al., 2004). In previous studies, low-dose benomyl treatment similarly reduced the distance between sister centromeres (Haase et al., 2012; Pearson et al., 2003). However, benomyl treatment did not increase the percentage of overlapping GFP spots and shortened spindle length, which is consistent with the mechanism of action of benomyl in promoting spindle and kinetochore microtubule depolymerization (Hochwagen et al., 2005). These data reveal that Taxol treatment suppresses microtubule dynamics and reduces both the time during which bipolar-attached sister centromeres are pulled apart and the distance that they are separated. Thus, the Taxol treatment reduces but does not eliminate microtubule-generated tension at sister centromeres and kinetochores.

For sister centromeres to move closer together yet remain attached, kinetochore microtubules should be on average longer in Taxol-treated spindles. To test this, we quantified GFP-labeled microtubules within metaphase spindles. As reported previously, the GFP signal is highest near the SPB, where spindle microtubules are nucleated, and decreases toward the spindle center as kinetochore microtubules terminate within each half-spindle (Figures 3F and 3G) (Shimogawa et al., 2006). Relative to the GFP signal near the SPB, this decrease occurs more gradually and remains higher toward the spindle center in Taxol-treated cells, which is consistent with longer kinetochore microtubules (Figure 3G). Budding yeast kinetochores bind just one microtubule, and thus cannot experience partial microtubule occupancy (Winey et al., 1995). Therefore, we have established conditions to reduce the tension at otherwise fully attached kinetochores.

Reduced Tension at Attached Kinetochores Produces a Delay in Anaphase Onset

To test the role of reduced kinetochore tension in delaying anaphase onset, we developed a “Cdc20 release assay.” Synchronized cells with preformed spindles were released into anaphase in the presence or absence of Taxol (Figure 4A). Chromosome separation is the key irreversible step in anaphase, and

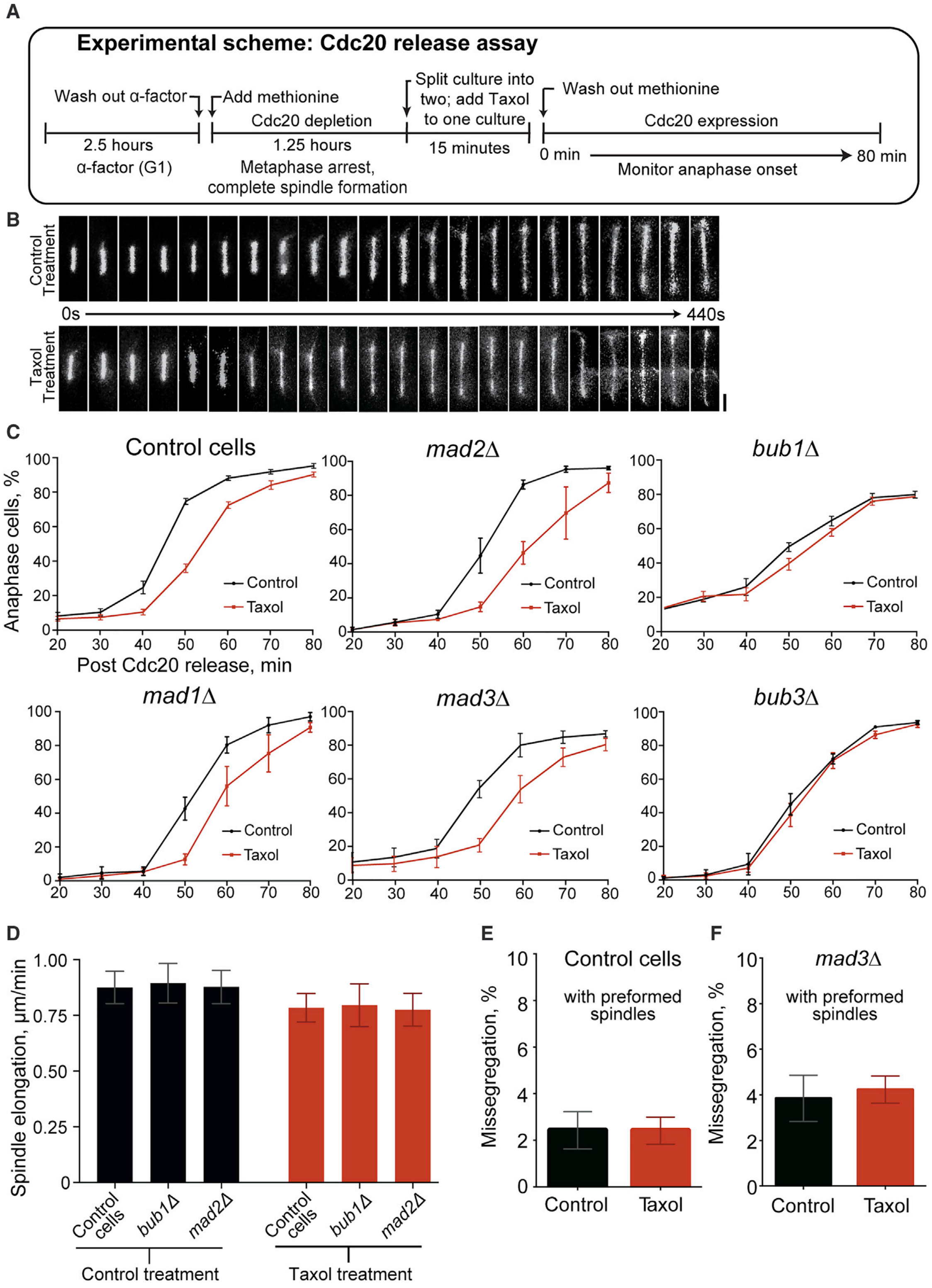

Figure 4. Reduced Tension at Attached Kinetochores Produces a Delay in Anaphase Onset that Requires Bub1 and Bub3, but Not Mad1, Mad2, or Mad3

(A) Experimental scheme of Cdc20 release assay.

(B) Anaphase spindle elongation viewed by fluorescent microtubules (GFP-Tub1). Images at 20-s intervals; bar, 2 μm.

(legend continued on next page)

under these conditions, Taxol treatment does not significantly perturb anaphase spindle elongation, which allowed us to score the timing of anaphase onset in individual cells (Figures 4B and S3A). In comparison to untreated cultures, cells in Taxol-treated cultures display a transient but reproducible delay in anaphase onset (Figure 4C). Although both Pds1-dependent and -independent mechanisms are known to delay anaphase onset, bulk analysis showed that Pds1 degradation occurred in both cultures, demonstrating that spindle elongation is concurrent with anaphase onset under both conditions (Figure S3B). In addition to scoring chromosome segregation by DAPI staining, we observed similar results monitoring spindle elongation in Spc29-mRFP cells (not shown). Taxol does not delay anaphase onset in drug-sensitized yeast that still harbors wild-type tubulin, showing that the delay in cells containing Taxol-sensitive tubulin is due to microtubule stabilization and not any secondary effect of Taxol (Figure S3C). We considered the possibility that a decrease in spindle elongation rate may delay chromosome separation. While not statistically significant, Taxol reduced the elongation rate ~15% (Figure 4D). At the average elongation rate, it would require 4.3 min for control spindles to elongate from 2 μm at anaphase onset to 6 μm, at which point telomere segregation is readily apparent (Straight et al., 1997). At the reduced rate, Taxol-treated spindles would require 5.1 min to reach 6 μm, which could account for only an ~1-min difference in the timing of chromosome separation. Thus, the observed delay does not result from a decreased spindle elongation rate.

Multiple lines of evidence indicate the Taxol-mediated delay in anaphase onset is not due to detached kinetochores. First, detached kinetochores produce a lengthy delay (Hoyt et al., 1991; Li and Murray, 1991). Second, the delay occurs in the entire population of cells. Thus, if it were due to detached kinetochores, every cell would possess at least one. However, Taxol does not significantly increase kinetochore detachment (Figure 2G). Third, detached kinetochores are predicted to increase chromosome missegregation, but missegregation is not increased when cells with preformed spindles are treated with Taxol (Figure 4E). Fourth, we monitored missegregation in cells lacking Mad3, which cannot delay anaphase onset in response to kinetochore detachment (Alexandru et al., 1999; Hardwick et al., 2000; Li and Murray, 1991; London and Biggins, 2014a). If kinetochores were detaching in every cell, even transiently, then they should missegregate at a higher frequency in *mad3Δ* cells. However, missegregation rates remain the same in control and Taxol-treated *mad3Δ* cells (Figure 4F). Thus, although Taxol produces a delay across the population of cells, evidence shows that the majority of cells do not possess unattached kinetochores. These results support the conclusion that reduced tension at attached kinetochores induces a delay in anaphase onset, independent of kinetochore detachment.

The Tension-Mediated Delay Requires the SAC Proteins Bub1 and Bub3, but Not Mad1, Mad2, or Mad3

We next sought to determine whether the SAC is involved in the tension-responsive delay in anaphase onset. The core SAC components Bub1, Bub3, Mad1, Mad2, and Mad3 are required for the response to detached kinetochores (Hoyt et al., 1991; Li and Murray, 1991; London and Biggins, 2014a). Thus, we tested whether they are needed for the tension-mediated delay revealed by the Cdc20 release assay. In cells lacking either Bub1 or Bub3, the Taxol-mediated delay is essentially lost, indicating that the tension-mediated mechanism requires these proteins (Figure 4C). The delay is unaffected by the loss of Mad1, Mad2, or Mad3 (Figure 4C). We considered whether altered spindle elongation rates may affect the apparent timing of chromosome separation in the mutant cells. However, the elongation rates in *bub1Δ* and *mad2Δ* cells are indistinguishable from those in control cells, regardless of Taxol treatment (Figure 4D). Thus, the timing of chromosome separation is not influenced by any potential effects of Bub1 or Mad2 on spindle elongation.

The fact that the Mad proteins are not needed for the Taxol-mediated delay provides additional evidence that this delay results from reduced tension. If the delay were due to detached kinetochores, then it should be abolished in the *mad* mutants (Li and Murray, 1991; London and Biggins, 2014a). In addition, chromosome missegregation is not increased when preformed spindles are treated with Taxol in *mad3Δ* cells, indicating that kinetochores remain attached during the Cdc20 release assay (Figure 4F). However, anaphase onset is delayed in these cells (Figure 4C). These results reveal that reduced tension at attached kinetochores induces a delay in anaphase onset that is mediated by a subset of the core SAC components.

SAC Components Mediate Distinct Responses to the Attachment and/or Tension Status at Kinetochores

In the Cdc20 release assay, reduced tension at kinetochores generates a delay that is significantly shorter than that reported for unattached kinetochores (Biggins and Murray, 2001; Hoyt et al., 1991; Li and Murray, 1991). In the G1 release assay, with unattached kinetochores present, the delay persists for at least 1 h (Figure 1G). In the Cdc20 release assay, *CDC20* is expressed from an inducible promoter. *CDC20* overexpression can override the SAC and promote premature anaphase onset (Hwang et al., 1998). Thus, it is possible that the duration of the tension-mediated delay in the Cdc20 release assay is shortened due to the altered kinetics of *CDC20* expression. To address this, we

(C) Timing of anaphase onset in Taxol-sensitive cells of the indicated genotype in the Cdc20 release assay (±30 μM Taxol). Time points represent means ± SEMs from 17 control cell, 3 *mad1Δ*, 3 *mad2Δ*, 4 *mad3Δ*, 5 *bub1Δ*, and 3 *bub3Δ* experiments; n = 100–200 cells per time point and drug condition for each experiment.
(D) Anaphase spindle elongation rates following release from Cdc20 depletion (±30 μM Taxol). Means ± SEMs for initial rapid elongation phase; n = 20 for each; differences were not statistically significant between control and Taxol treatments nor between genotypes in either treatment.
(E and F) Percentage of missegregation of *CEN1*-GFP marked chromosomes in control (E) and *mad3Δ* (F) cells following anaphase in the Cdc20 release assay. Means ± SEMs from 8 control and 4 *mad3Δ* experiments. For control cells, n = 113, 124, 133, 127, 98, 149, 110, and 117 (untreated) and 109, 133, 128, 151, 109, 127, 113, and 139 (Taxol treated). For *mad3Δ* cells, n = 131, 147, 134, and 91 (untreated) and 147, 137, 116, and 111 (Taxol treated). Control and Taxol treatments are not statistically significant for both (E) and (F).
See also Figure S3.

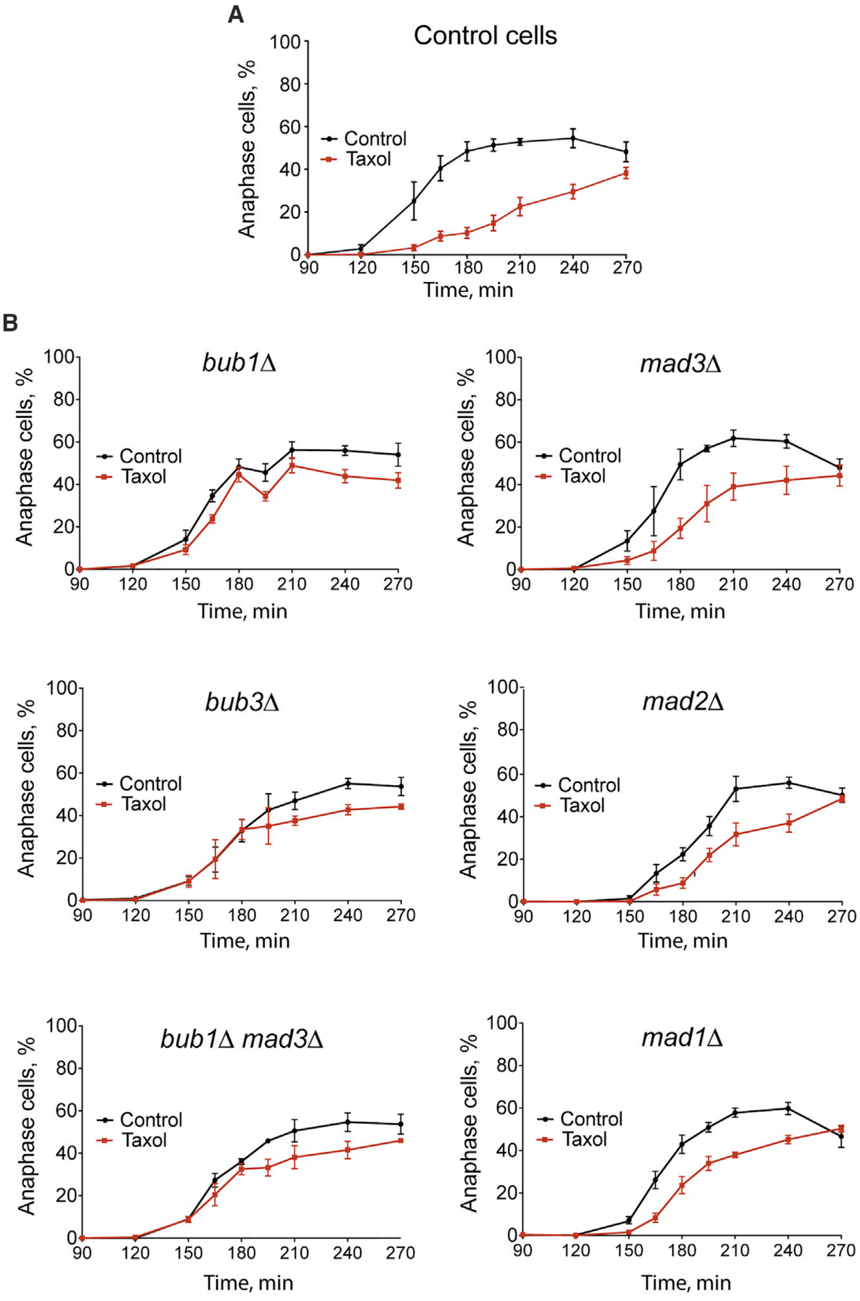

Figure 5. Components of the Spindle Assembly Checkpoint Mediate Distinct Responses to the Attachment and/or Tension Status at Kinetochores

Timing of anaphase onset in Taxol-sensitive cells of the indicated genotype in the G1 release assay (±30 μM Taxol).

(A) Control cells delay anaphase onset for ∼1 h during Taxol treatment, which produces both unattached and low-tension kinetochores.

(B) *bub1Δ* and *bub3Δ* cells, which do not display tension-mediated delays in the Cdc20 release assay (Figure 4C), fail to delay anaphase onset during Taxol treatment in the G1 release assay. *mad1Δ*, *mad2Δ*, and *mad3Δ* cells, which do display tension-mediated delays in the Cdc20 release assay (Figure 4C), also delay anaphase onset during Taxol treatment in the G1 release assay.

Time points represent means ± SEMs from (A) 6 and (B) >3 experiments, with n = 100–200 cells per time point and drug condition for each experiment. See also Figure S4.

sought to determine the length of the tension-mediated delay with *CDC20* expression under endogenous regulation.

When Taxol is present during spindle assembly, anaphase onset is normally delayed with both unattached and low-tension kinetochores present (Figure 5A). Both Bub1 and Bub3 are required for the SAC-mediated delay from unattached kinetochores (Hoyt et al., 1991; London and Biggins, 2014a). Our results with preformed spindles show that they are also required to delay anaphase onset due to reduced tension (Figure 4C). Thus, in the G1 release assay, *bub1Δ* and *bub3Δ* cells should fail to respond to either the unattached or low-tension status of kinetochores. Consistent with this prediction, the Taxol-induced hour-long delay is abolished in both *bub1Δ* and *bub3Δ* cells (Figure 5B).

Mad1, Mad2, and Mad3 are required for the SAC response to unattached kinetochores (Li and Murray, 1991; London and Biggins, 2014a). However, results from the Cdc20 release assay, where Taxol is added after spindle assembly, show that Mad1, Mad2, and Mad3 are not required for the tension-mediated delay (Figure 4C). Thus, although cells lacking these proteins cannot delay due to the unattached kinetochore status, they should retain the ability to delay anaphase onset due to the low-tension status in the G1 release assay. Consistent with this prediction, *mad1Δ*, *mad2Δ*, and *mad3Δ* cells delay anaphase onset ∼20 min during Taxol treatment (Figure 5B). As with control cells, Taxol does not affect the timing of cell-cycle initiation in *bub1Δ* or *mad3Δ* cells (Figure S4). In addition, Taxol does not delay anaphase onset in *bub1Δ mad3Δ* cells, indicating that the delay in *mad3Δ* cells is mediated through Bub1 and not a secondary effect of *MAD3* deletion (Figure 5B).

Taxol treatment following G1 release induces a mitotic delay that is distinct from the canonical SAC response to unattached kinetochores and is mechanistically consistent with the tension-mediated delay observed with preformed spindles. Overall, the data segregate the core SAC proteins into two classes. The first class, Bub1 and Bub3, function in both the attachment- and tension-mediated mechanisms, while the second class, Mad1,

Mad2, and Mad3, are not required to transiently delay anaphase onset in the presence of tensionless kinetochores.

Yeast Cells Lacking Bub or Mad Proteins Display Differential Sensitivity to Taxol

Our results predict that the SAC mutants should have different sensitivities to Taxol. At very low Taxol concentrations, bipolar kinetochore attachments will likely be assembled in a timely manner, and cells will not depend on the SAC for viability. We reasoned that at moderately low concentrations, however, the establishment of proper attachments will become slightly delayed. Although cells lacking Mad1, Mad2, or Mad3 cannot respond to unattached kinetochores, the tension-mediated delay mechanism still provides up to ∼20 min to achieve proper attachments. However, because cells lacking Bub1 or Bub3 respond to neither unattached nor tensionless kinetochores, they are not granted time to complete the bipolar attachment process and lose viability at these concentrations. At higher Taxol concentrations, microtubules become increasingly stabilized and more time is required for spindle assembly than the tension-mediated mechanism alone can provide. At this point, the longer delay induced by unattached kinetochores, present in control cells but absent in cells lacking Mad1, Mad2, or Mad3, becomes essential for viability.

To test this prediction, we monitored the growth of Taxol-sensitive cells lacking SAC components on medium containing the drug. Relative to cells with an intact SAC, *mad1Δ*, *mad2Δ*, and *mad3Δ* cells display increased Taxol sensitivity (Figure 6A). As predicted, both *bub1Δ* and *bub3Δ* cells are notably more sensitive than the *mad* mutants (Figure 6A). This indicates that Bub1 and Bub3 do not function only in a single linear pathway with the Mad proteins in response to Taxol treatment. Rather than an additive effect, *bub1Δ mad3Δ* cells display sensitivity that is similar to *bub1Δ* cells (Figure 6A). This is consistent with the idea that all five proteins share a common function, but that Bub1 and Bub3 play an additional role required to protect cells from the tension-reducing effects of Taxol treatment.

Microtubule-Generated Tension and the SAC

What the role is of low tension at kinetochores in delaying mitotic progression is a long-standing question. Using a Taxol-sensitive yeast model, we show that reduced tension at attached kinetochores delays anaphase onset. Unlike experiments with unpaired chromosomes (Shonn et al., 2000; Stern and Murray, 2001), the Taxol treatment of preformed spindles used here does not significantly perturb bipolar spindle organization or anaphase elongation. This allowed the assessment of kinetochore attachment pre- and post-anaphase, which is not altered by Taxol treatment. Our results reveal that Bub1 and Bub3 function to delay anaphase onset in response to low tension, independent of kinetochore detachment (Figure 6B).

The tension-specific delay mechanism is distinct from that of unattached kinetochores, yet it could operate within a common framework. At unattached kinetochores, the core SAC proteins assemble in a stepwise manner to generate the mitotic checkpoint complex (MCC) that sequesters Cdc20 and inhibits the APC (Figure 6C) (London and Biggins, 2014a). Initially, phosphorylation of Spc105/KLN1 by Mps1 recruits Bub3-Bub1 to the kinetochore. Next, Bub3-BubR1/Mad3 is recruited, although in budding yeast, Mad3 does not appear to localize to the kinetochore (Gillett et al., 2004). Mad1 and Mad2 are then recruited and facilitate MCC formation. In this hierarchical process, Bub1 and Bub3 function before the Mad proteins (Figure 6C). In addition to our data, other evidence indicates that Bub1 and Bub3 behave differently than Mad1 and Mad2 in the context of attached versus unattached kinetochores. In budding yeast, Bub1 and Bub3 normally localize to kinetochores early in mitosis, yet Mad1 and Mad2 do so only when microtubule-kinetochore attachments are absent (Gillett et al., 2004). *Xenopus* Bub1 remains associated with kinetochores that have bound microtubules, whereas Mad1 and Mad2 do not (Sharp-Baker and Chen, 2001). Bub1 and Bub3 also co-purify with yeast kinetochores, whereas Mad1 does so only after treatment with the microtubule destabilizer benomyl (London and Biggins, 2014b). These observations are consistent with our data showing that Bub1 and Bub3 act in response to low tension, independent of the Mad proteins. We speculate that early events involving Bub1 and Bub3 may be tension sensitive, whereas the lack of microtubule binding facilitates Mad-dependent MCC formation (Figure 6D).

The tension-specific mechanism is mediated by Bub1 and Bub3, but it is independent of the MCC. In HeLa cells Bub1 and Polo-like kinase Plk1 function together to phosphorylate Cdc20 and inhibit APC activation, separately from MCC-mediated inhibition (Jia et al., 2016). In yeast, Bub1 has been linked to the tension-dependent localization of pericentromeric Shugoshin (Sgo1) (Nerusheva et al., 2014), and there is evidence that Sgo1, Slk19, and the protein phosphatase 2A (PP2A) phosphatase regulatory subunit Cdc55 can delay anaphase onset by inhibiting Separase independent of Pds1 degradation (Clift et al., 2009; Lianga et al., 2018). It will be important to determine whether the phosphorylation of Cdc20 by Bub1 is linked to tension status and whether the tension-specific delay mechanism is independent of Pds1 degradation and/or uses Sgo1, Slk19, or Cdc55.

Whether microtubule-generated tension silences the SAC is under debate. Evidence obtained in several organisms suggests that tension may serve to inactivate SAC signaling (Jin and Wang, 2013; Jin et al., 2017; Maresca and Salmon, 2009; Uchida et al., 2009; Wan et al., 2009). The super-resolution light microscopy of kinetochore components in *Drosophila* and human HeLa cells indicates that increased stretch within the kinetochore is correlated with SAC inactivation (Maresca and Salmon, 2009; Uchida et al., 2009; Wan et al., 2009). Using similar techniques, it was shown that the SAC can be silenced without full intra-kinetochore stretch (Etemad et al., 2015; Tauchman et al., 2015). Moreover, integrated light and electron microscopy revealed that super-resolution measurements of intra-kinetochore stretch are sensitive to variable shape and organizational changes in response to tension, fixation techniques, and visualization methods (Magidson et al., 2016). We used a readout of kinetochore tension that assesses microtubule-generated tension across sister kinetochores. This shows that overall tension is reduced by Taxol treatment, but it does not specifically reveal the status of intra-kinetochore stretch.

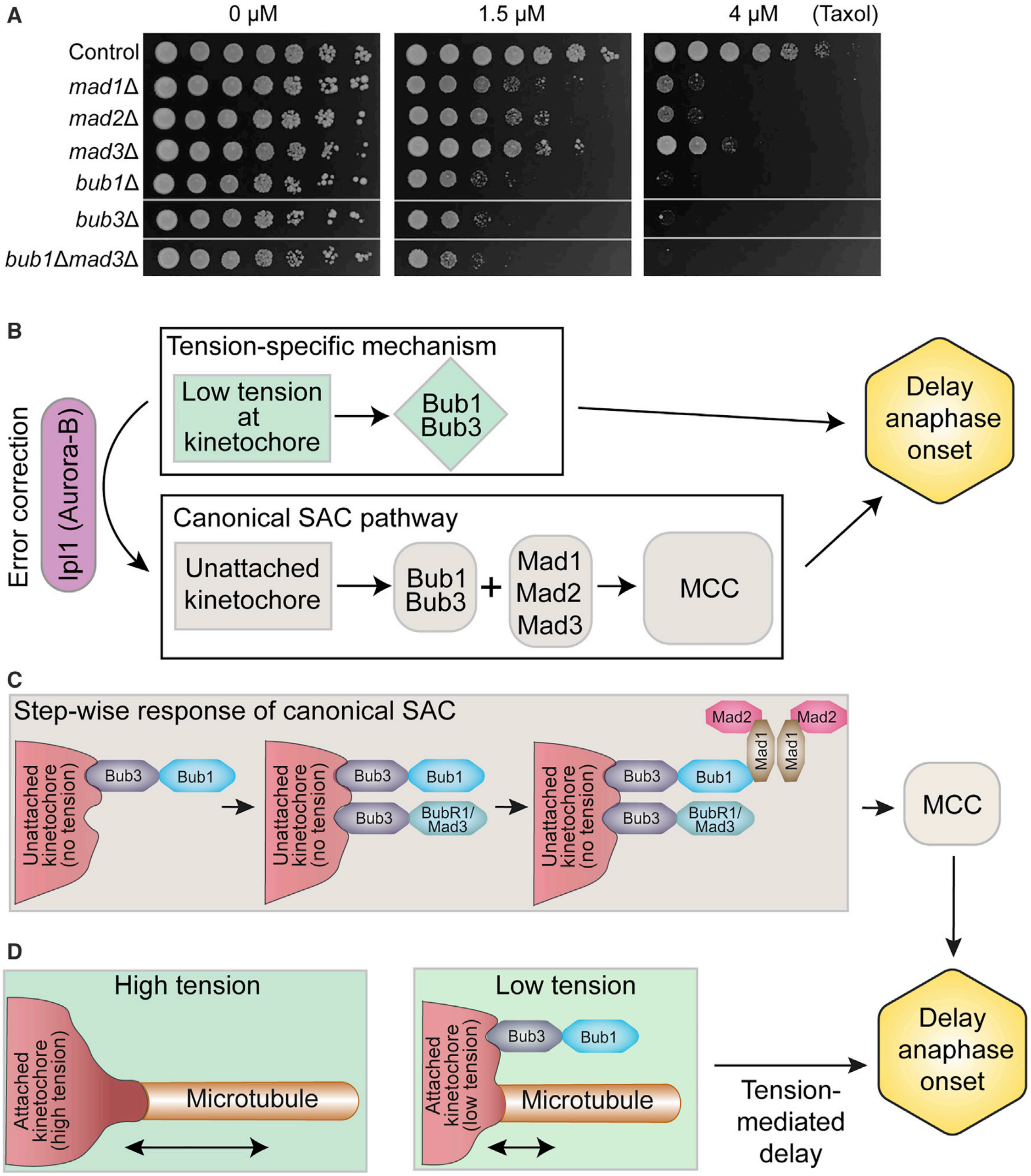

Figure 6. Cells Lacking Spindle Assembly Checkpoint Components Display Differential Sensitivity to Taxol

(A) Serial dilutions of Taxol-sensitive cells of the indicated genotype were spotted onto plates containing the indicated concentration of Taxol. All of the strains were grown on the same plate; gray lines denote where intervening lanes were cropped.

(B) Low tension at attached kinetochores delays anaphase onset via Bub1 and Bub3 but is independent of the mitotic checkpoint complex (MCC; consisting of Mad2, Bub3, BubR1/Mad3, and Cdc20).

(C) Stepwise SAC response to unattached kinetochores. Note that Mad3 appears to not localize to kinetochores in budding yeast (London and Biggins, 2014a).

(D) Speculative model for how the Bub1- and Bub3-dependent tension-specific response could operate within the context of the canonical SAC.

We find that reducing tension in preformed spindles, after bipolar attachments have been established and experienced tension, still delays anaphase onset. This also induces delay in *mad* mutants, in which SAC activation and the subsequent need for silencing is inhibited. Thus, despite any potential role in SAC silencing, our data show that a lack of tension generates a signal that postpones mitotic progression.

It is unclear why Ipl1-mediated error correction does not detach kinetochores in preformed spindles treated with Taxol. Because Taxol does not completely eliminate tension under

these conditions (Figures 3C–3E), sufficient tension may remain to avoid error correction. Alternatively, Taxol-mediated microtubule stabilization may directly oppose microtubule detachment. Although we cannot absolutely exclude the possibility that Taxol-treatment alters the nature of end-on kinetochore attachments, we did not mutate kinetochore proteins nor perturb intercentromeric structure. Moreover, multiple lines of evidence show that kinetochores maintain bipolar attachments in our assays, and data from drug-sensitized cells harboring wild-type tubulin confirm that the delay results directly from microtubule stabilization.

The tension-specific delay is shorter than that observed for unattached kinetochores. Transient delays have been seen in various organisms under conditions that reduce tension (Biggins and Murray, 2001; Maresca and Salmon, 2009; O'Connell et al., 2008; Suzuki et al., 2016; Uchida et al., 2009; Zasadil et al., 2014). In human retinal pigmented epithelial (RPE1) cells, Taxol treatment in late metaphase produces a modest delay in most cells, and prolonged delay is correlated with Mad2 recruitment to detached kinetochores (Magidson et al., 2016). Work in yeast using a fluorescence resonance energy transfer (FRET)-based tension sensor built into the Ndc80 kinetochore protein revealed that the removal of the microtubule-binding tail of Ncd80 results in reduced tension and increased metaphase duration (Suzuki et al., 2016). Together with our data, these results support the conclusion that a lack of tension generates a signal that can delay but not prevent anaphase onset in the absence of kinetochore detachment. Consistent with this, the treatment of HeLa cells with low-dose nocodazole to suppress microtubule-generated tension during metaphase inhibited but did not abolish APC activity, leading to anaphase onset (Uchida et al., 2009). It remains possible, however, that reducing tension produces a graded response, as seen with the number of unattached kinetochores (Dick and Gerlich, 2013). In this regard, removal of a portion of the Ndc80 tail produces an intermediate reduction in FRET-measured tension and a modest increase in metaphase duration (Suzuki et al., 2016). In addition, rendering all kinetochores tensionless via unpaired chromosomes generates extended delays, although it is difficult to exclude the contribution from unattached kinetochores with this approach (Barnhart et al., 2011; Biggins and Murray, 2001; Indjeian et al., 2005; King et al., 2007; Lee and Spencer, 2004; Makrantoni and Stark, 2009; Stern and Murray, 2001).

A transient delay in mitotic progression when tension is low may serve to provide cells with additional time to establish a sufficiently robust bipolar spindle or to detach tensionless connections and subsequently respond to the newly unattached kinetochore. HeLa cells require at least 5 min for a freshly detached kinetochore to inhibit the APC (Dick and Gerlich, 2013). Thus, a detachment that occurs <5 min before chromosome separation risks missegregation. Perhaps the tension-mediated delay signal helps alleviate this vulnerable period. Notably, unattached kinetochores are also tensionless, and how their tension-based signaling contributes to the overall length of delay they produce remains to be determined. One challenge will be to separate the tension- and attachment-mediated signaling output. Doing so will provide valuable data for understanding how cells use these two mechanisms to safeguard their genome.

STAR★METHODS

Detailed methods are provided in the online version of this paper and include the following:

- KEY RESOURCES TABLE
- CONTACT FOR REAGENT AND RESOURCE SHARING
- EXPERIMENTAL MODEL AND SUBJECT DETAILS
  - Yeast Strains
- METHOD DETAILS
  - Media Specifications
  - Spotting Assays
  - G1 Release Assay
  - Preformed Metaphase Spindles by Cdc20 Depletion
  - Cdc20 Release Assay
  - Microscopy
  - Imaging and Analysis of Metaphase Spindles in Fixed Cells
  - Imaging and Analysis of Live Cells
  - Fluorescence-Activated Cell Sorting (FACS)
  - Western Blotting
- QUANTIFICATION AND STATISTICAL ANALYSIS

SUPPLEMENTAL INFORMATION

Supplemental Information can be found online at https://doi.org/10.1016/j.celrep.2019.03.027.

ACKNOWLEDGMENTS

We thank S. Biggins, T. Davis, C. Janke, P. Meluh, E. Muller, K. Nasmyth, D. Pellman, and F. Uhlmann for useful reagents and tools, and are grateful to S. Biggins and E. Nsamba for helpful feedback. This work was supported in part by Iowa State University, an NSF grant (MCB-1846262) to M.L.G., and an NIH predoctoral training fellowship (T32GM007183) to K.G.P.

AUTHOR CONTRIBUTIONS

M.L.G. conceived the project; M.L.G., K.G.P., and S.J.A. designed the research and constructed the reagents; and M.L.G., K.G.P., S.D., S.J.A., A.R.B., P.S.R., and A.B. performed the experiments, analyzed the data, prepared the figures, and wrote the manuscript.

DECLARATION OF INTERESTS

The authors declare no competing interests.

Received: July 17, 2017
Revised: May 18, 2018
Accepted: March 8, 2019
Published: April 9, 2019

REFERENCES

Alexandru, G., Zachariae, W., Schleiffer, A., and Nasmyth, K. (1999). Sister chromatid separation and chromosome re-duplication are regulated by different mechanisms in response to spindle damage. EMBO J. *18*, 2707–2721.

Barnes, G., Louie, K.A., and Botstein, D. (1992). Yeast proteins associated with microtubules in vitro and in vivo. Mol. Biol. Cell *3*, 29–47.

Barnhart, E.L., Dorer, R.K., Murray, A.W., and Schuyler, S.C. (2011). Reduced Mad2 expression keeps relaxed kinetochores from arresting budding yeast in mitosis. Mol. Biol. Cell *22*, 2448–2457.

Biggins, S., and Murray, A.W. (2001). The budding yeast protein kinase Ipl1/Aurora allows the absence of tension to activate the spindle checkpoint. Genes Dev. *15*, 3118–3129.

Bode, C.J., Gupta, M.L., Jr., Reiff, E.A., Suprenant, K.A., Georg, G.I., and Himes, R.H. (2002). Epothilone and paclitaxel: unexpected differences in promoting the assembly and stabilization of yeast microtubules. Biochemistry *41*, 3870–3874.

Clift, D., Bizzari, F., and Marston, A.L. (2009). Shugoshin prevents cohesin cleavage by PP2A(Cdc55)-dependent inhibition of separase. Genes Dev. *23*, 766–780.

Dick, A.E., and Gerlich, D.W. (2013). Kinetic framework of spindle assembly checkpoint signalling. Nat. Cell Biol. *15*, 1370–1377.

Entwistle, R.A., Rizk, R.S., Cheng, D.M., Lushington, G.H., Himes, R.H., and Gupta, M.L., Jr. (2012). Differentiating between models of epothilone binding to microtubules using tubulin mutagenesis, cytotoxicity, and molecular modeling. ChemMedChem *7*, 1580–1586.

Etemad, B., Kuijt, T.E.F., and Kops, G.J.P.L. (2015). Kinetochore-microtubule attachment is sufficient to satisfy the human spindle assembly checkpoint. Nat. Commun. *6*, 8987.

Foland, T.B., Dentler, W.L., Suprenant, K.A., Gupta, M.L., Jr., and Himes, R.H. (2005). Paclitaxel-induced microtubule stabilization causes mitotic block and apoptotic-like cell death in a paclitaxel-sensitive strain of Saccharomyces cerevisiae. Yeast *22*, 971–978.

Fuchs, D.A., and Johnson, R.K. (1978). Cytologic evidence that taxol, an antineoplastic agent from Taxus brevifolia, acts as a mitotic spindle poison. Cancer Treat. Rep. *62*, 1219–1222.

Gillett, E.S., Espelin, C.W., and Sorger, P.K. (2004). Spindle checkpoint proteins and chromosome-microtubule attachment in budding yeast. J. Cell Biol. *164*, 535–546.

Gordon, D.J., Resio, B., and Pellman, D. (2012). Causes and consequences of aneuploidy in cancer. Nat. Rev. Genet *13*, 189–203.

Goshima, G., and Yanagida, M. (2000). Establishing biorientation occurs with precocious separation of the sister kinetochores, but not the arms, in the early spindle of budding yeast. Cell *100*, 619–633.

Gupta, M.L., Jr., Bode, C.J., Georg, G.I., and Himes, R.H. (2003). Understanding tubulin-Taxol interactions: mutations that impart Taxol binding to yeast tubulin. Proc. Natl. Acad. Sci. USA *100*, 6394–6397.

Gupta, K., Bishop, J., Peck, A., Brown, J., Wilson, L., and Panda, D. (2004). Antimitotic antifungal compound benomyl inhibits brain microtubule polymerization and dynamics and cancer cell proliferation at mitosis, by binding to a novel site in tubulin. Biochemistry *43*, 6645–6655.

Haase, J., Stephens, A., Verdaasdonk, J., Yeh, E., and Bloom, K. (2012). Bub1 kinase and Sgo1 modulate pericentric chromatin in response to altered microtubule dynamics. Curr. Biol. *22*, 471–481.

Hardwick, K.G., Johnston, R.C., Smith, D.L., and Murray, A.W. (2000). MAD3 encodes a novel component of the spindle checkpoint which interacts with Bub3p, Cdc20p, and Mad2p. J. Cell Biol. *148*, 871–882.

He, X., Asthana, S., and Sorger, P.K. (2000). Transient sister chromatid separation and elastic deformation of chromosomes during mitosis in budding yeast. Cell *101*, 763–775.

Hochwagen, A., Wrobel, G., Cartron, M., Demougin, P., Niederhauser-Wiederkehr, C., Boselli, M.G., Primig, M., and Amon, A. (2005). Novel response to microtubule perturbation in meiosis. Mol. Cell. Biol. *25*, 4767–4781.

Hoyt, M.A., Totis, L., and Roberts, B.T. (1991). S. cerevisiae genes required for cell cycle arrest in response to loss of microtubule function. Cell *66*, 507–517.

Hsu, J.-M., Huang, J., Meluh, P.B., and Laurent, B.C. (2003). The yeast RSC chromatin-remodeling complex is required for kinetochore function in chromosome segregation. Mol. Cell. Biol. *23*, 3202–3215.

Hwang, L.H., Lau, L.F., Smith, D.L., Mistrot, C.A., Hardwick, K.G., Hwang, E.S., Amon, A., and Murray, A.W. (1998). Budding yeast Cdc20: a target of the spindle checkpoint. Science *279*, 1041–1044.

Indjeian, V.B., Stern, B.M., and Murray, A.W. (2005). The centromeric protein Sgo1 is required to sense lack of tension on mitotic chromosomes. Science *307*, 130–133.

Jia, L., Li, B., and Yu, H. (2016). The Bub1-Plk1 kinase complex promotes spindle checkpoint signalling through Cdc20 phosphorylation. Nat. Commun. *7*, 10818.

Jin, F., and Wang, Y. (2013). The signaling network that silences the spindle assembly checkpoint upon the establishment of chromosome bipolar attachment. Proc. Natl. Acad. Sci. USA *110*, 21036–21041.

Jin, F., Bokros, M., and Wang, Y. (2017). Premature Silencing of the Spindle Assembly Checkpoint Is Prevented by the Bub1-H2A-Sgo1-PP2A Axis in *Saccharomyces cerevisiae*. Genetics *205*, 1169–1178.

Jordan, M.A., Toso, R.J., Thrower, D., and Wilson, L. (1993). Mechanism of mitotic block and inhibition of cell proliferation by taxol at low concentrations. Proc. Natl. Acad. Sci. USA *90*, 9552–9556.

Kelling, J., Sullivan, K., Wilson, L., and Jordan, M.A. (2003). Suppression of centromere dynamics by Taxol in living osteosarcoma cells. Cancer Res. *63*, 2794–2801.

Khodjakov, A., and Pines, J. (2010). Centromere tension: a divisive issue. Nat. Cell Biol. *12*, 919–923.

King, J.M., and Nicklas, R.B. (2000). Tension on chromosomes increases the number of kinetochore microtubules but only within limits. J. Cell Sci. *113*, 3815–3823.

King, E.M.J., Rachidi, N., Morrice, N., Hardwick, K.G., and Stark, M.J.R. (2007). Ipl1p-dependent phosphorylation of Mad3p is required for the spindle checkpoint response to lack of tension at kinetochores. Genes Dev. *21*, 1163–1168.

Krenn, V., and Musacchio, A. (2015). The Aurora B Kinase in Chromosome Bi-Orientation and Spindle Checkpoint Signaling. Front. Oncol. *5*, 225.

Lee, M.S., and Spencer, F.A. (2004). Bipolar orientation of chromosomes in Saccharomyces cerevisiae is monitored by Mad1 and Mad2, but not by Mad3. Proc. Natl. Acad. Sci. USA *101*, 10655–10660.

Li, R., and Murray, A.W. (1991). Feedback control of mitosis in budding yeast. Cell *66*, 519–531.

Li, X., and Nicklas, R.B. (1995). Mitotic forces control a cell-cycle checkpoint. Nature *373*, 630–632.

Lianga, N., Doré, C., Kennedy, E.K., Yeh, E., Williams, E.C., Fortinez, C.M., Wang, A., Bloom, K.S., and Rudner, A.D. (2018). Cdk1 phosphorylation of Esp1/Separase functions with PP2A and Slk19 to regulate pericentric Cohesin and anaphase onset. PLoS Genet. *14*, e1007029.

Lim, H.H., Goh, P.Y., and Surana, U. (1998). Cdc20 is essential for the cyclosome-mediated proteolysis of both Pds1 and Clb2 during M phase in budding yeast. Curr. Biol. *8*, 231–234.

London, N., and Biggins, S. (2014a). Signalling dynamics in the spindle checkpoint response. Nat. Rev. Mol. Cell Biol. *15*, 736–747.

London, N., and Biggins, S. (2014b). Mad1 kinetochore recruitment by Mps1-mediated phosphorylation of Bub1 signals the spindle checkpoint. Genes Dev. *28*, 140–152.

Luchniak, A., Fukuda, Y., and Gupta, M.L., Jr. (2013). Structure-function analysis of yeast tubulin. Methods Cell Biol. *115*, 355–374.

Magidson, V., He, J., Ault, J.G., O'Connell, C.B., Yang, N., Tikhonenko, I., McEwen, B.F., Sui, H., and Khodjakov, A. (2016). Unattached kinetochores rather than intrakinetochore tension arrest mitosis in taxol-treated cells. J. Cell Biol. *212*, 307–319.

Makrantoni, V., and Stark, M.J.R. (2009). Efficient chromosome biorientation and the tension checkpoint in Saccharomyces cerevisiae both require Bir1. Mol. Cell. Biol. *29*, 4552–4562.

Maresca, T.J., and Salmon, E.D. (2009). Intrakinetochore stretch is associated with changes in kinetochore phosphorylation and spindle assembly checkpoint activity. J. Cell Biol. *184*, 373–381.

Maresca, T.J., and Salmon, E.D. (2010). Welcome to a new kind of tension: translating kinetochore mechanics into a wait-anaphase signal. J. Cell Sci. *123*, 825–835.

McEwen, B.F., Heagle, A.B., Cassels, G.O., Buttle, K.F., and Rieder, C.L. (1997). Kinetochore fiber maturation in PtK1 cells and its implications for the mechanisms of chromosome congression and anaphase onset. J. Cell Biol. *137*, 1567–1580.

Michaelis, C., Ciosk, R., and Nasmyth, K. (1997). Cohesins: chromosomal proteins that prevent premature separation of sister chromatids. Cell *91*, 35–45.

Murray, A.W. (2011). A brief history of error. Nat. Cell Biol. *13*, 1178–1182.

Nerusheva, O.O., Galander, S., Fernius, J., Kelly, D., and Marston, A.L. (2014). Tension-dependent removal of pericentromeric shugoshin is an indicator of sister chromosome biorientation. Genes Dev. *28*, 1291–1309.

Nezi, L., and Musacchio, A. (2009). Sister chromatid tension and the spindle assembly checkpoint. Curr. Opin. Cell Biol. *21*, 785–795.

Nicklas, R.B., and Ward, S.C. (1994). Elements of error correction in mitosis: microtubule capture, release, and tension. J. Cell Biol. *126*, 1241–1253.

Nicklas, R.B., Ward, S.C., and Gorbsky, G.J. (1995). Kinetochore chemistry is sensitive to tension and may link mitotic forces to a cell cycle checkpoint. J. Cell Biol. *130*, 929–939.

Nicklas, R.B., Waters, J.C., Salmon, E.D., and Ward, S.C. (2001). Checkpoint signals in grasshopper meiosis are sensitive to microtubule attachment, but tension is still essential. J. Cell Sci. *114*, 4173–4183.

O'Connell, C.B., Loncarek, J., Hergert, P., Kourtidis, A., Conklin, D.S., and Khodjakov, A. (2008). The spindle assembly checkpoint is satisfied in the absence of interkinetochore tension during mitosis with unreplicated genomes. J. Cell Biol. *183*, 29–36.

Pearson, C.G., Maddox, P.S., Salmon, E.D., and Bloom, K. (2001). Budding yeast chromosome structure and dynamics during mitosis. J. Cell Biol. *152*, 1255–1266.

Pearson, C.G., Maddox, P.S., Zarzar, T.R., Salmon, E.D., and Bloom, K. (2003). Yeast kinetochores do not stabilize Stu2p-dependent spindle microtubule dynamics. Mol. Biol. Cell *14*, 4181–4195.

Piatti, S., Lengauer, C., and Nasmyth, K. (1995). Cdc6 is an unstable protein whose de novo synthesis in G1 is important for the onset of S phase and for preventing a 'reductional' anaphase in the budding yeast Saccharomyces cerevisiae. EMBO J. *14*, 3788–3799.

Pinsky, B.A., Kung, C., Shokat, K.M., and Biggins, S. (2006). The Ipl1-Aurora protein kinase activates the spindle checkpoint by creating unattached kinetochores. Nat. Cell Biol. *8*, 78–83.

Rieder, C.L., Schultz, A., Cole, R., and Sluder, G. (1994). Anaphase onset in vertebrate somatic cells is controlled by a checkpoint that monitors sister kinetochore attachment to the spindle. J. Cell Biol. *127*, 1301–1310.

Rieder, C.L., Cole, R.W., Khodjakov, A., and Sluder, G. (1995). The checkpoint delaying anaphase in response to chromosome monoorientation is mediated by an inhibitory signal produced by unattached kinetochores. J. Cell Biol. *130*, 941–948.

Rizk, R.S., Discipio, K.A., Proudfoot, K.G., and Gupta, M.L., Jr. (2014). The kinesin-8 Kip3 scales anaphase spindle length by suppression of midzone microtubule polymerization. J. Cell Biol. *204*, 965–975.

Rose, M.D., Winston, F., and Hieter, P. (1990). Methods in Yeast Genetics (Cold Spring Harbor Laboratory Press).

Schiff, P.B., and Horwitz, S.B. (1980). Taxol stabilizes microtubules in mouse fibroblast cells. Proc. Natl. Acad. Sci. USA *77*, 1561–1565.

Shannon, K.B., Canman, J.C., and Salmon, E.D. (2002). Mad2 and BubR1 function in a single checkpoint pathway that responds to a loss of tension. Mol. Biol. Cell *13*, 3706–3719.

Sharp-Baker, H., and Chen, R.H. (2001). Spindle checkpoint protein Bub1 is required for kinetochore localization of Mad1, Mad2, Bub3, and CENP-E, independently of its kinase activity. J. Cell Biol. *153*, 1239–1250.

Shimogawa, M.M., Graczyk, B., Gardner, M.K., Francis, S.E., White, E.A., Ess, M., Molk, J.N., Ruse, C., Niessen, S., Yates, J.R., 3rd., et al. (2006). Mps1 phosphorylation of Dam1 couples kinetochores to microtubule plus ends at metaphase. Curr. Biol. *16*, 1489–1501.

Shimogawa, M.M., Widlund, P.O., Riffle, M., Ess, M., and Davis, T.N. (2009). Bir1 is required for the tension checkpoint. Mol. Biol. Cell *20*, 915–923.

Shonn, M.A., McCarroll, R., and Murray, A.W. (2000). Requirement of the spindle checkpoint for proper chromosome segregation in budding yeast meiosis. Science *289*, 300–303.

Siegel, J.J., and Amon, A. (2012). New insights into the trouble of aneuploidy. Annu. Rev. Cell Dev. Biol. *28*, 189–214.

Skoufias, D.A., Andreassen, P.R., Lacroix, F.B., Wilson, L., and Margolis, R.L. (2001). Mammalian mad2 and bub1/bubR1 recognize distinct spindle-attachment and kinetochore-tension checkpoints. Proc. Natl. Acad. Sci. USA *98*, 4492–4497.

Stern, B.M., and Murray, A.W. (2001). Lack of tension at kinetochores activates the spindle checkpoint in budding yeast. Curr. Biol. *11*, 1462–1467.

Straight, A.F., Marshall, W.F., Sedat, J.W., and Murray, A.W. (1997). Mitosis in living budding yeast: anaphase A but no metaphase plate. Science *277*, 574–578.

Suzuki, A., Badger, B.L., Haase, J., Ohashi, T., Erickson, H.P., Salmon, E.D., and Bloom, K. (2016). How the kinetochore couples microtubule force and centromere stretch to move chromosomes. Nat. Cell Biol. *18*, 382–392.

Tanaka, T., Fuchs, J., Loidl, J., and Nasmyth, K. (2000). Cohesin ensures bipolar attachment of microtubules to sister centromeres and resists their precocious separation. Nat. Cell Biol. *2*, 492–499.

Tanaka, T.U., Rachidi, N., Janke, C., Pereira, G., Galova, M., Schiebel, E., Stark, M.J.R., and Nasmyth, K. (2002). Evidence that the Ipl1-Sli15 (Aurora kinase-INCENP) complex promotes chromosome bi-orientation by altering kinetochore-spindle pole connections. Cell *108*, 317–329.

Tauchman, E.C., Boehm, F.J., and DeLuca, J.G. (2015). Stable kinetochore-microtubule attachment is sufficient to silence the spindle assembly checkpoint in human cells. Nat. Commun. *6*, 10036.

Uchida, K.S.K., Takagaki, K., Kumada, K., Hirayama, Y., Noda, T., and Hirota, T. (2009). Kinetochore stretching inactivates the spindle assembly checkpoint. J. Cell Biol. *184*, 383–390.

Uhlmann, F., Wernic, D., Poupart, M.A., Koonin, E.V., and Nasmyth, K. (2000). Cleavage of cohesin by the CD clan protease separin triggers anaphase in yeast. Cell *103*, 375–386.

Wan, X., O'Quinn, R.P., Pierce, H.L., Joglekar, A.P., Gall, W.E., DeLuca, J.G., Carroll, C.W., Liu, S.-T., Yen, T.J., McEwen, B.F., et al. (2009). Protein architecture of the human kinetochore microtubule attachment site. Cell *137*, 672–684.

Waters, J.C., Chen, R.H., Murray, A.W., and Salmon, E.D. (1998). Localization of Mad2 to kinetochores depends on microtubule attachment, not tension. J. Cell Biol. *141*, 1181–1191.

Winefield, R.D., Entwistle, R.A., Foland, T.B., Lushington, G.H., and Himes, R.H. (2008). Differences in paclitaxel and docetaxel interactions with tubulin detected by mutagenesis of yeast tubulin. ChemMedChem *3*, 1844–1847.

Winey, M., Mamay, C.L., O'Toole, E.T., Mastronarde, D.N., Giddings, T.H., Jr., McDonald, K.L., and McIntosh, J.R. (1995). Three-dimensional ultrastructural analysis of the Saccharomyces cerevisiae mitotic spindle. J. Cell Biol. *129*, 1601–1615.

Yeh, E., Skibbens, R.V., Cheng, J.W., Salmon, E.D., and Bloom, K. (1995). Spindle dynamics and cell cycle regulation of dynein in the budding yeast, Saccharomyces cerevisiae. J. Cell Biol. *130*, 687–700.

Zasadil, L.M., Andersen, K.A., Yeum, D., Rocque, G.B., Wilke, L.G., Tevaarwerk, A.J., Raines, R.T., Burkard, M.E., and Weaver, B.A. (2014). Cytotoxicity of paclitaxel in breast cancer is due to chromosome missegregation on multipolar spindles. Sci. Transl. Med. *6*, 229ra43.

STAR★METHODS

KEY RESOURCES TABLE

REAGENT or RESOURCE	SOURCE	IDENTIFIER
Antibodies		
Mouse Anti-c-Myc	Core hybridoma facility	Clone 9E10
Mouse Anti-beta Actin	Abcam	Cat# ab8224; RRID:AB_449644
Sheep Anti-mouse IgG HRP Conjugated	GE Healthcare	Cat# NA931-1ML; RRID:AB_772210
Chemicals, Peptides, and Recombinant Proteins		
Bacto peptone	BD Biosciences	Cat# 211677
Experimental Models: Organisms/Strains		
S. cerevisiae: Strain Background: S288C; see Table S1	N/A	S288C; BY4741
Software and Algorithms		
GraphPad Prism 6	GraphPad Software	RRID:SCR_002798
SlideBook 6	Intelligent Imaging Innovations, Inc.	RRID:SCR_014300
ImageJ	https://imagej.net/Welcome; http://fiji.sc	RRID:SCR_003070
MetaMorph Microscopy Automation & Image Analysis Software	Molecular Devices, LLC.	RRID:SCR_002368
MATLAB 2016b	MathWorks	RRID:SCR_001622
Fluorcal and Calcmate Scripts for MATLAB	Shimogawa et al., 2009	N/A

CONTACT FOR REAGENT AND RESOURCE SHARING

Further information and requests for resources and reagents should be directed to and will be fulfilled by the Lead Contact, Mohan Gupta (mgupta@iastate.edu).

EXPERIMENTAL MODEL AND SUBJECT DETAILS

Yeast Strains

Yeast strains are of S288C background and described in Table S1. The original Taxol-sensitive yeast tubulin contains five substitutions (A19K, T23V, G26D, N227H, Y270F) (Gupta et al., 2003) and does not perturb microtubule dynamics *in vivo* (Entwistle et al., 2012). Here we utilized the '*tub2-25'* allele with four substitutions (A19K, T23V, G26D, Y270F) that retains full Taxol sensitivity (Winefield et al., 2008). The drug-sensitized background was created by *pdr1*Δ *pdr3*Δ *erg6*Δ. The Taxol-sensitive background is *tub2-25 pdr1Δ pdr3Δ erg6Δ*. Genetic deletions were introduced into control cells by fragment-mediated homologous recombination or by genetic crossing. Deletions were sequence-verified, then crossed into the Taxol-sensitive background and re-verified to generate the Taxol-sensitive mutant strains. Yeast media and genetic techniques were performed as described previously (Rose et al., 1990). Details of strain construction are available upon request. Taxol is the brand name for paclitaxel (Bristol-Myers Squibb, Princeton, NJ).

METHOD DETAILS

Media Specifications

We found Taxol to be more effective at inhibiting cell proliferation on agar plates than in liquid media. Taxol effectiveness in liquid media was increased by adding 0.02% methylcellulose (cP15) to SC or YPD media from a sterile 2% stock. However, Taxol in liquid media containing 0.02% methylcellulose remained less effective relative to agar plates. Thus, unless otherwise specified experiments in liquid media were conducted using 30 μM Taxol. To ensure that drug-sensitized yeast (*pdr1*Δ *pdr3*Δ *erg6*Δ) were not affected by environmental components or detergents, all glassware was cleaned by filling with glass-distilled water and autoclaving, then emptied and autoclaved again to sterilize and dry. Media was also made with glass-distilled water. YPD and SC drop-out media were made according to standard recipes. However, we found the growth of drug-sensitized yeast in YPD can be influenced by peptone from various sources, particularly at room temperature. After comparative testing we found BD-brand Bactopeptone

(BD Biosciences Cat# 211677) to be most consistent between control and drug-sensitized yeast. We found no growth variation attributable to components of SC media or other ingredients of YPD.

Spotting Assays

2X YPD (filter-sterilized) was mixed with 2X agar (hot; autoclaved) and stirred. Before cooling, 35 mL was poured into a 50 mL conical tube and Taxol was added from a 1 mM stock in DMSO. The tube was closed and inverted 5 times to mix before pouring the plate. All plates contained the same amount of DMSO regardless of Taxol concentration. Plates were allowed to dry under a laminar flow hood and used within one day. Serial dilutions (10x) were prepared from 2-day saturated cultures, 2.2 μL of each dilution was spotted onto the plates, and the plates were incubated at 24°C for 5 days.

G1 Release Assay

*MAT***a** cells grown to mid-log phase (30°C) in SC media were spun down and resuspended in SC media containing 100 μM alpha factor. Following a 2.5-hour incubation at 30°C, the zero-minute time point was collected. The G1-synchronized culture was then split in two, washed three times with water and resuspended in 5 mL SC media containing 0.02% methylcellulose (cP15) and DMSO ± 30 μM Taxol (final from 1 mM stock in DMSO). The cultures were then maintained on the bench and gently mixed by hand every 10 min. Samples were removed and fixed in ice cold 70% ethanol at 60, 90, 120, 150, 165, 180, 195, 210, 240, and 270 min. Fixed cells were washed twice in PBS and stained with 50 ng/ml DAPI. To image cells, 10-12 z-plane DIC and DAPI images were collected at 0.5 μm intervals. To quantify images, cells were categorized based on DNA and cell morphology; single cells with unseparated DNA, lacking a bud were considered "unbudded"; those with a bud $<^3/_4$ the diameter of the mother were "small budded metaphase," while those with a bud $\geq ^3/_4$ the diameter of the mother were "big budded metaphase"; budded cells whose DNA had visibly separated into two distinct masses were considered "anaphase" cells; and finally in the later time points, cells with DNA morphology indicating they had undergone anaphase and had also grown a new bud, were considered "anaphase re-budded." Budding index was calculated as the number of small + large-budded cells out of the total number of cells. Percent anaphase cells was calculated as the number of anaphase + anaphase re-budded cells out of the total number of cells. Images were coded and all G1 release assays were scored blinded to both cell genotype and Taxol status.

Preformed Metaphase Spindles by Cdc20 Depletion

*MAT***a** strains were modified to place the Anaphase Promoting Complex co-activator, Cdc20, under the methionine-inducible *MET3* promoter (Uhlmann et al., 2000). Cells were maintained and grown to mid-log phase (30°C) in SC -Met media. Cells were transferred to SC -Met with 100 μM alpha-factor for 2.5 hours at 30°C to synchronize in G1. These cells were washed three times in 1 mL water and resuspended in SC -Met containing 0.02% methylcellulose and supplemented with 0.2 mg/ml methionine, to suppress Cdc20 production. After 75 min at 30°C, > 90% of cells were arrested in metaphase, and DMSO ± 30 μM Taxol (final from 1 mM stock in DMSO) was added to the cultures. 15 min later cells were either imaged live or fixed with 3.7% formaldehyde and imaged within 24 h.

Cdc20 Release Assay

Cells were prepared as in the Cdc20 arrest (above), however, following the 15 min incubation ± Taxol, cells were quickly washed three times in 1 mL water (to remove methionine) and resuspended in 5 mL SC -Met containing 0.02% methylcellulose and DMSO ± 30 μM Taxol (final from 1 mM stock in DMSO). In the absence of methionine, cells produce Cdc20 and release from arrest. Cultures were maintained on the bench and gently mixed by hand every 10 min. Samples were removed and fixed in ice cold 70% ethanol every 10 min over 80 min. Cells were washed 2 times with PBS and nuclei were stained with 50 ng/ml DAPI. For imaging, 10-12 z-plane DIC and DAPI images were collected at 0.5 μm intervals. Images were coded and Cdc20 release assays and missegregation assays were scored blinded to both cell genotype and Taxol status. To quantify images, cells were categorized based on DNA and cell morphology; single cells lacking a bud were considered "single cells," cells with a small or large bud whose DNA had not yet begun to separate were considered "metaphase," and budded cells whose DNA had visibly separated into two distinct masses were considered "anaphase" cells. To score chromosome missegregation, cells in which *CEN1* was marked by tetO/tetR-GFP were used in the Cdc20 release assay and imaged with 20 z-planes spaced 0.3 μm apart. At 50 and 70 min post Cdc20 release, cells with elongated spindles were scored for whether the two tetO/tetR-GFP foci were segregated correctly into the mother and daughter cells.

Microscopy

Spindle poles were visualized with endogenous Spc29-mRFP, and microtubules with an exogenous copy of GFP-Tub1 integrated at the URA3 locus. Centromeres were visualized by tetO/tetR-GFP arrays; 224 tandem repeats of tetO were integrated 228 bp 3′ of CDEIII on chromosome 1 using plasmid pPM290 (Hsu et al., 2003) and visualized with TetR-YFP or TetR-GFP (Michaelis et al., 1997). Live and fixed cell imaging was performed on a Carl Zeiss AxioImager M2 microscope with a piezoelectric-driven Z-stage and a cooled CCD camera (CoolSNAP HQ^2; Photometrics). Images were obtained with a 63 × 1.4 NA Plan Apochromat objective and Semrock filters using SlideBook software (Intelligent Imaging Innovations, Inc.). Live cell imaging of spindle elongation was also performed on a Nikon ECLIPSE-Ti inverted microscope equipped with a Ti-ND6-PFS Perfect Focus Unit and a Yokogawa CSU-XI spinning disk confocal scanner. Images were captured on an Andor iXon3 897 EMCCD camera using a Nikon CFI Plan

Apo VC 60X 1.2 NA water immersion objective and MetaMorph Microscopy Automation & Image Analysis Software (Molecular Devices, LLC.).

Imaging and Analysis of Metaphase Spindles in Fixed Cells

20 z-plane images spaced 0.3 μm apart were captured in the RFP (SPB) and either YFP or GFP (*CEN1*-tetO/tetR) channels. Images were coded and then scored in blinded fashion with respect to Taxol and nocodazole status. For analysis, only spindles fully contained within the image stacks were used. Slidebook software was used to measure distances between the outer edges of SPBs or sister centromeres in 3-dimensions across the z stack. To determine the distance between the centroids of separated sister centromeres, the x-y-z coordinates representing the centroid of each centromere fluorescence signal was determined using Slidebook software and used to calculate the 3-dimensional distance. To score centromere and/or kinetochore attachment to metaphase spindles, a square was generated by extending lines from each pole at 45° angles to the spindle axis. Centromeres and/or kinetochores were considered attached if both centromere foci were within this square.

Imaging and Analysis of Live Cells

To measure spindle elongation rates, cells released from metaphase arrest in the Cdc20 release assay were applied to a glass microscope slide and the coverslip sealed with Valap (Luchniak et al., 2013). Cells were imaged at 20 s intervals using 10 z-plane images spaced 0.75 μm apart. Elongation rates were determined as described previously (Rizk et al., 2014). Anaphase spindles in yeast undergo a rapid elongation from ~2 μm in metaphase to ~6 μm (Yeh et al., 1995), at which length chromosome and telomere separation is readily apparent (Straight et al., 1997). The potential delay in observed chromosome separation due to reduced spindle elongation rate was calculated as the difference in time required for control and Taxol-treated spindles to elongate from 2 to 6 μm: Elongation Time Difference = (4 μm / 0.79 μm/min Taxol elongation rate) – (4 μm / 0.93 μm/min control elongation rate) = 0.76 min.

GFP-labeled microtubule fluorescence intensity in metaphase spindles was determined using the semiautomated MATLAB scripts "fluorcal" and "calcmate" (Shimogawa et al., 2009). Cells arrested by Cdc20 depletion were imaged with a 100 × 1.4 NA Plan Apochromat objective using 20 z-plane images spaced 0.2 μm apart. With spindles between 1.6 – 2 μm long, GFP fluorescence intensity in maximum z-projection images was determined along the length of the spindle with a 10-pixel wide line, using the adjacent 2 pixels along each side as background. Spindle fluorescence intensity data were normalized to 24 pixels, plus an additional 20% extending beyond each end of the spindle (pushout), and each pixel value was then calculated as a fraction of total spindle fluorescence (Shimogawa et al., 2009). In Figure 3G the maximum intensities are normalized to 1.0 for comparison.

To measure astral microtubule dynamics cells were imaged at room temperature on an upright microscope at 10 s intervals in SC media containing 0.02% methylcellulose and DMSO ± 30 μM Taxol. Microtubule length was calculated as the average of two independent measurements of the 3-dimensional length at each time point and parameters of dynamic instability were calculated essentially as previously described (Entwistle et al., 2012). Periods of polymerization and depolymerization were defined as a line through at least four points (30 s) with a length change $\geq$ 0.4 μm and an $R^2 \geq 0.84$. Periods of attenuation were scored as > 30 s with net length changes < ± 0.02 μm. Brief periods that did not fit these criteria were omitted. Catastrophes were defined as the transition into depolymerization from either polymerization or attenuation, and the time spent polymerizing and attenuated was used to determine frequency. Rescues were scored as transition from depolymerization into polymerization or attenuation, and only time depolymerizing was used to calculate frequency.

To image centromeres on metaphase spindles, cells were imaged at room temperature in SC -Met media, 0.2mg/ml methionine, 0.02% methylcellulose and DMSO ± 30 μM Taxol. Images were captured in the GFP (*CEN1*-tetO/tetR) and RFP (SPB) channels at 2-3 s intervals. For analysis, time-lapse images of individual cells were cropped out and coded before centromere separation and breathing was scored blinded with regard to Taxol treatment. Only images in which both spindle poles were in focus were used to score centromere separation as either a single or two separated fluorescent foci. Transitions per minute, or 'breathing' was calculated as the number of transitions from unseparated to separated, and vice versa, over time. To generate kymographs, at each time point the centroid of one SPB was located using the 'find foci' tool and then translated (along with other channels) to the center of the image. The centroid of the second SPB was then located and the image rotated to align both SPBs on the x axis. Kymographs were then generated using ImageJ (National Institutes of Health).

Fluorescence-Activated Cell Sorting (FACS)

Cultures were processed as described for the G1 release and Cdc20 release assays. Cells were removed at each time point, fixed with ice cold 70% ethanol and treated overnight at 37°C in 1 mL of 0.20 mg/ml DNAase-free RNAaseA in 1 M Tris (pH 7.4). Samples were stained with propidium iodide in PBS buffer and sonicated briefly (5x1 s at 50% setting) prior to analysis on a BD FACSCanto II. Data were analyzed using Cytobank.

Western Blotting

To blot Pds1-18myc, aliquots of cultures were removed during the G1 or Cdc20 release assays and placed on ice. Whole-cell protein extracts were prepared by NaOH treatment followed by lysis in Laemmli buffer heated to 95°C for 10 min. Clarified samples were

electrophoresed on a 6% Tris-glycine gel and blotted using a semi-dry apparatus. Blots were developed with anti-myc – clone 9E10 from mouse, anti-beta actin – clone ab8224 from mouse (Abcam), and anti-mouse IgG HRP conjugated from sheep (GE Healthcare) using ECL detection (RPN3243; GE Healthcare).

QUANTIFICATION AND STATISTICAL ANALYSIS

p values were determined by unpaired Student's t test using Prism 6 software (* $p \leq 0.05$, ** $p \leq 0.01$, *** $p \leq 0.001$, **** $p \leq 0.0001$, ns = not statistically significant). SD and SEM refer to standard deviation and standard error of the mean, respectively. Statistical details including number of replicates and sample sizes can be found in the corresponding figure legends.

Cell Reports, Volume *27*

Supplemental Information

Checkpoint Proteins Bub1 and Bub3 Delay Anaphase Onset in Response to Low Tension Independent of Microtubule-Kinetochore Detachment

Kathleen G. Proudfoot, Samuel J. Anderson, Sandeep Dave, Angela R. Bunning, Pallavi Sinha Roy, Abesh Bera, and Mohan L. Gupta Jr.

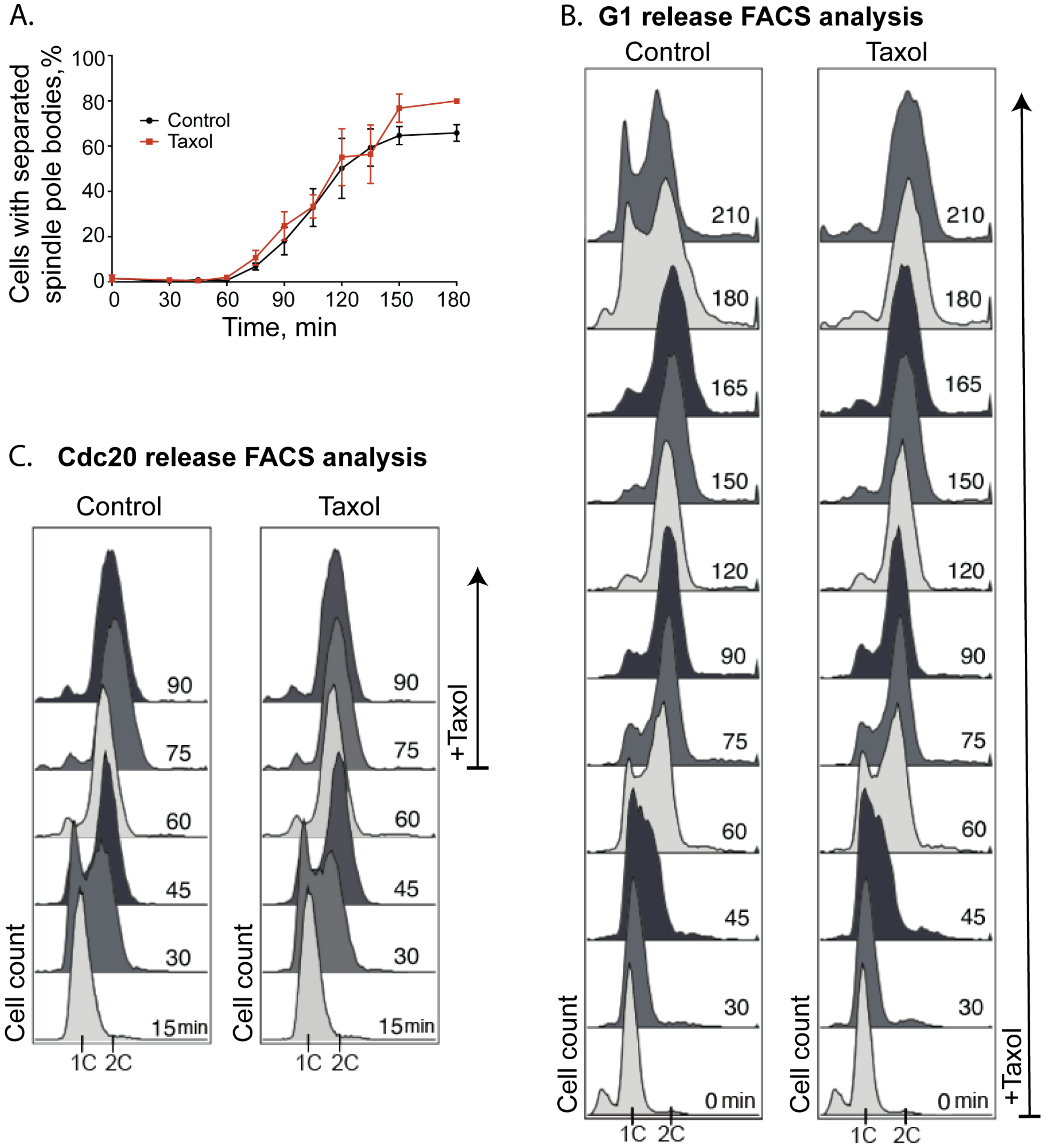

Figure S1. Kinetics of spindle pole body (SPB) separation and DNA replication in the presence and absence of Taxol. Related to Figure 1, Figure 2 and Figure 3. (A) SPB separation during the G1 release assay. Taxol-sensitive cells synchronized in G1 (alpha-factor) were released into the cell cycle and split into sister cultures either with or without 30 μM Taxol. During the ensuing cell cycle SPB separation was monitored by Spc29-mRFP. Experimental scheme of the G1 release assay described in Figure 1E. Plotted values represent the mean ± SEM from 3 experiments, with n = 100-200 cells scored per time point and drug condition for each experiment. (B) DNA replication measured by flow cytometry during the G1 release assay. Taxol was added to the indicated culture at 0 min. DNA replication occurs with similar timing in both cultures and Taxol inhibits cell cycle progression at 180 min. (C) DNA replication measured by flow cytometry during the Cdc20 release assay. Taxol was added to the indicated culture at 75 min. DNA replication occurs with similar timing in both cultures and is essentially complete by 75 min. In (A) and (B) changing colors serve only to create contrast between overlapping histograms.

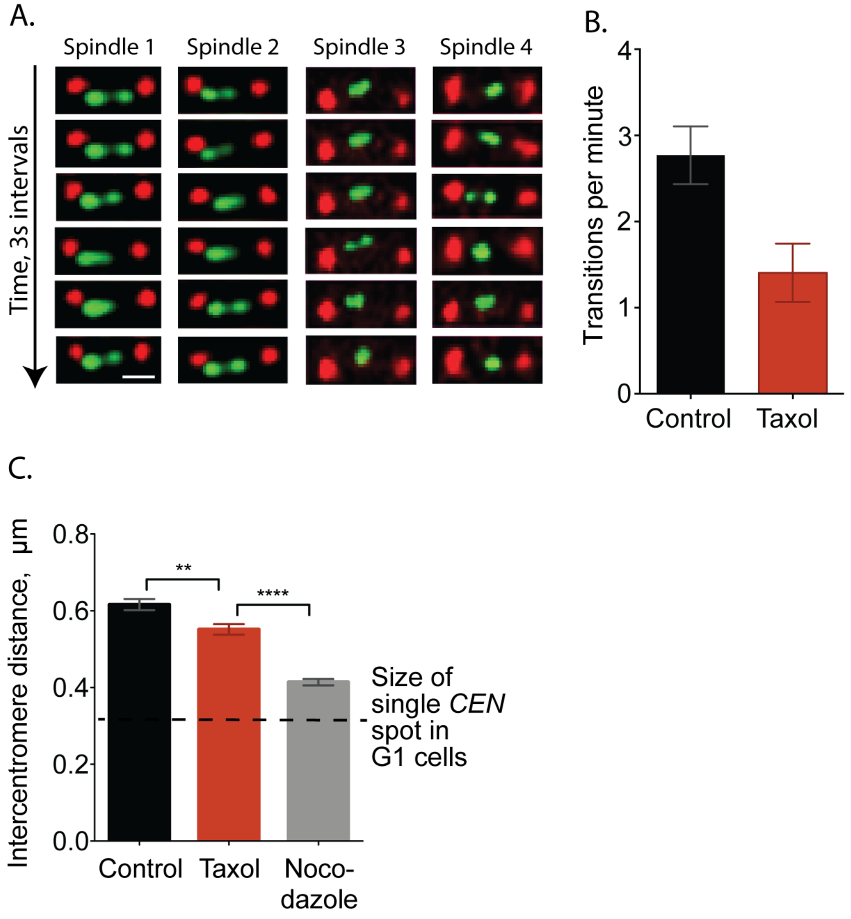

Figure S2. Taxol treatment reduces tension at attached kinetochores. Related to Figure 3. Taxol-sensitive cells were released from G1 synchronization (alpha-factor) into methionine-containing media to hold cells in metaphase with fully formed spindles (Cdc20 depletion). Cultures were split, treated with DMSO ± 30 µM Taxol or 15 µg/ml nocodazole for 15 min, and imaged either live (A-B) or fixed (C). Experimental scheme described in Figure 2A. Sister centromeres were localized by *CEN1*-tetO/tetR-GFP imaging. (A) Representative examples of brief centromere breathing events in metaphase spindles. Spindle poles are red (Spc29-mRFP, outer spots); *CEN1*-GFP is green; time interval between images is 3s; bar = 1 µm. (B) Frequency of transition between either one or two visible *CEN1*-GFP foci. Data represents mean ± SEM observed in individual live cells; n = 31 cells for both; $p = 0.0058$. (C) Distance between the outer edges of *CEN1*-GFP foci in fixed cells (both separated and unseparated). The size of a single, unreplicated *CEN1*-GFP focus in G1 cells is 0.32 ± 0.004 µm (dashed line; n = 170). In nocodazole treated cells, the distance between sister centromeres essentially represents the minimum size of two adjacent *CEN1*-GFP foci in which the majority are tensionless. Mean ± SEM from 4, 4 and 3 experiments for control (n = 178, 156, 145, 169), Taxol (n = 179, 178, 153, 98), and nocodazole (n = 86, 84, 100) treated cells, respectively. $p = 0.0015$ for control versus Taxol, and $p < 0.0001$ for Taxol versus nocodazole treated cells. p values were determined by unpaired student's t-test (*$p \leq 0.05$, **$p \leq 0.01$, ***$p \leq 0.001$, ****$p \leq 0.0001$).

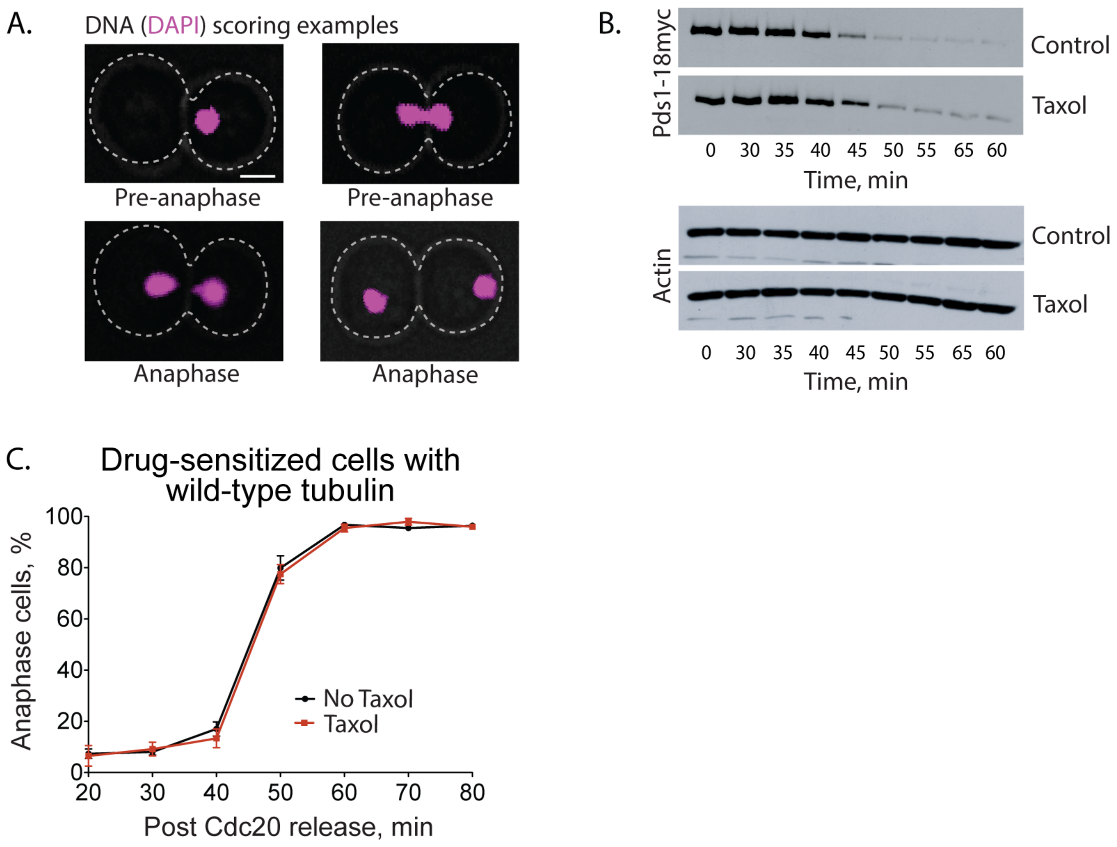

Figure S3. Taxol-induced anaphase delay is dependent on Taxol-tubulin binding. Related to Figure 4. In the Cdc20 release assay, cells synchronized in G1 (alpha-factor) were released into media containing methionine to hold cells in metaphase with fully formed spindles (Cdc20 depletion). Cultures were split, treated with DMSO ± 30 µM Taxol and released from metaphase. Experimental scheme of the Cdc20 release assay described in Figure 4A. (A) Representative images of DAPI-stained cells monitored during the Cdc20 release assay. Bar = 2 µm. (B) Western blot of Pds1-18myc degradation in control and Taxol-treated cells during the Cdc20 release assay. Actin was blotted as a loading control. (C) Timing of anaphase onset in drug-sensitized cells with wild-type yeast tubulin monitored by the Cdc20 release assay (± 30 µM Taxol). Values represent mean ± SEM from 3 experiments, with n = 100-200 cells scored per time point and drug condition for each experiment.

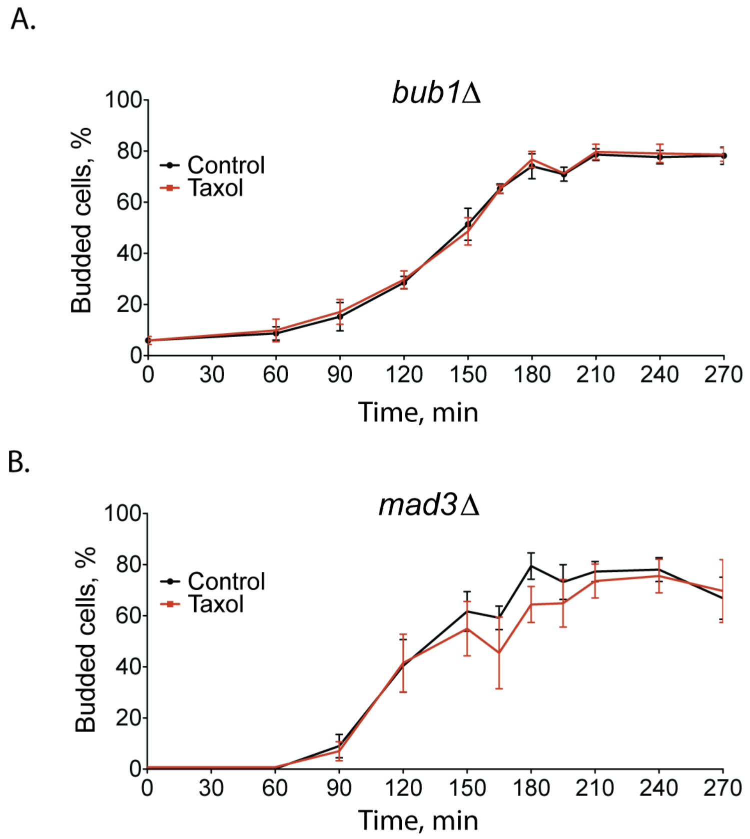

Figure S4. Budding index of cells monitored in the G1 release assay. Related to Figure 5. Taxol-sensitive (A) *bub1*Δ or (B) *mad3*Δ cells synchronized in G1 (alpha-factor) were released into the cell cycle and split into sister cultures either with or without 30 μM Taxol. During the ensuing cell cycle, bud emergence was monitored by cell morphology. Experimental scheme of the G1 release assay described in Figure 1E. Plotted values represent the mean ± SEM from 7 experiments for *bub1*Δ and 6 for *mad3*Δ cells, with n = 100-200 cells scored per time point and drug condition for each experiment.

Table S1. Yeast strains used in this study. Related to STAR Methods.

Strain	Genotype
MGY50	*MAT***a**, *TUB2, ura3-52*, *his3Δ200*, *leu2Δ1*, *trp1Δ63*
MGY980	*MAT***a**, *tub2-25* (A19K-T23V-G26D-Y270F), *erg6::HIS3, pdr1::hygB, pdr3::NAT, his3Δ200, ura3-52, trp1Δ63, cen1::URA3-CEN3-tetO, pURA3-tetR-YFP-ADHterm-LEU2, SPC29-mRFP-KanR, pMET3-3xHA-CDC20-TRP1*
MGY1293	*MAT***a**, *tub2-25* (A19K-T23V-G26D-Y270F), *erg6::HIS3, pdr1::hygB, pdr3::NAT, his3Δ200, leu2Δ1, ura3-52, trp1Δ63, pMET3-3xHA-CDC20-TRP1*
MGY1315	*MAT***a**, *tub2-25* (A19K-T23V-G26D-Y270F), *erg6::HIS3, pdr1::hygB, pdr3::NAT, his3Δ200, leu2Δ1, ura3-52, trp1Δ63, pMET3-3xHA-CDC20-TRP1, bub1Δ::KanR*
MGY1392	*MAT***a**, *tub2-25* (A19K-T23V-G26D-Y270F), *erg6::HIS3, pdr1::hygB, pdr3::NAT, his3Δ200, leu2Δ1, ura3-52, trp1Δ63, pMET3-3xHA-CDC20-TRP1, PDS1-18myc-LEU2*
MGY1399	*MAT***a**, *tub2-25* (A19K-T23V-G26D-Y270F), *erg6::TRP1, pdr1::hygB, pdr3::NAT, his3Δ200, leu2Δ1, trp1Δ63, GFP-TUB1-URA3*
MGY1428	*MAT***a**, *TUB2, erg6::HIS3, pdr1::hygB, pdr3::NAT, his3Δ200, leu2Δ1, ura3-52, trp1Δ63, pMET3-3xHA-CDC20-TRP1*
MGY1508	*MAT***a**, *tub2-25* (A19K-T23V-G26D-Y270F), *erg6::HIS3, pdr1::hygB, pdr3::NAT, his3Δ200, leu2Δ1, ura3-52, trp1Δ63, pMET3-3xHA-CDC20-TRP1, mad3Δ::KanR*
MGY1693	*MAT***a**, *tub2-25* (A19K-T23V-G26D-Y270F), *erg6::HIS3, pdr1::hygB, pdr3::NAT, his3Δ200, leu2Δ1, ura3-52, trp1Δ63, pMET3-3xHA-CDC20-TRP1, mad1Δ::KanR*
MGY1739	*MAT***a**, *tub2-25* (A19K-T23V-G26D-Y270F), *erg6::HIS3, pdr1::hygB, pdr3::NAT, his3Δ200, leu2Δ1, ura3-52, trp1Δ63, pMET3-3xHA-CDC20-TRP1, bub3Δ::KanR*
MGY1749	*MAT***a**, *tub2-25* (A19K-T23V-G26D-Y270F), *erg6::HIS3, pdr1::hygB, pdr3::NAT, his3Δ200, leu2Δ1, ura3-52, trp1Δ63, cen1::URA3-CEN3-tetO, 5'NLS-tetR-GFP-ADHterm-LEU2, SPC29-mRFP-KanR, pMET3-3xHA-CDC20-TRP1*
MGY1830	*MAT***a**, *tub2-25* (A19K-T23V-G26D-Y270F), *erg6::HIS3, pdr1::hygB, pdr3::NAT, his3Δ200, leu2Δ1, ura3-52, trp1Δ63, pMET3-3xHA-CDC20-TRP1, mad2Δ::KanR*
MGY1872	*MAT***a**, *TUB2, erg6::TRP1, pdr1::hygB, pdr3::NAT, his3Δ200, leu2Δ1, ura3-52, trp1Δ63*
MGY2103	*MAT***a**, *tub2-25* (A19K-T23V-G26D-Y270F), *erg6::TRP1, pdr1::hygB, pdr3::NAT, his3Δ200, leu2Δ1, ura3-52, trp1Δ63, bub3Δ::KanR*
MGY2127	*MAT***a**, *tub2-25* (A19K-T23V-G26D-Y270F), *erg6::HIS3, pdr1::hygB, pdr3::NAT, his3Δ200, leu2Δ1, ura3-52, trp1Δ63, cen1::URA3-CEN3-tetO, 5'NLS-tetR-GFP-ADHterm-LEU2, pMET3-3xHA-CDC20-TRP1, mad3Δ::KanR*
MGY2128	*MAT***a**, *tub2-25* (A19K-T23V-G26D-Y270F), *erg6::TRP1, pdr1::hygB, pdr3::NAT, his3Δ200, leu2Δ1, ura3-52, trp1Δ63, bub1Δ::KanR*
MGY2133	*MAT***a**, *tub2-25* (A19K-T23V-G26D-Y270F), *erg6::TRP1, pdr1::hygB, pdr3::NAT, his3Δ200, leu2Δ1, ura3-52, trp1Δ63, mad1Δ::KanR*
MGY2134	*MAT***a**, *tub2-25* (A19K-T23V-G26D-Y270F), *erg6::TRP1, pdr1::hygB, pdr3::NAT, his3Δ200, leu2Δ1, ura3-52, trp1Δ63, mad2Δ::KanR*
MGY2136	*MAT***a**, *tub2-25* (A19K-T23V-G26D-Y270F), *erg6::TRP1, pdr1::hygB, pdr3::NAT, his3Δ200, leu2Δ1, ura3-52, trp1Δ63, mad3Δ::KanR*
MGY2139	*MAT***a**, *tub2-25* (A19K-T23V-G26D-Y270F), *erg6::TRP1, pdr1::hygB, pdr3::NAT, his3Δ200, leu2Δ1, ura3-52, trp1Δ63, mad3Δ::KanR, bub1Δ::KanR*
MGY2150	*MAT***a**, *tub2-25* (A19K-T23V-G26D-Y270F), *erg6::TRP1, pdr1::hygB, pdr3::NAT, his3Δ200, leu2Δ1, ura3-52, trp1Δ63*
MGY2226	*MAT***a**, *tub2-25* (A19K-T23V-G26D-Y270F), *erg6::HIS3, pdr1::hygB, pdr3::NAT, his3Δ200, leu2Δ1, ura3-52, trp1Δ63, pMET3-3xHA-CDC20-TRP1, GFP-TUB1-URA3*
MGY2227	*MAT***a**, *tub2-25* (A19K-T23V-G26D-Y270F), *erg6::HIS3, pdr1::hygB, pdr3::NAT, his3Δ200, leu2Δ1, ura3-52, trp1Δ63, pMET3-3xHA-CDC20-TRP1, bub1Δ::KanR, GFP-TUB1-URA3*
MGY2228	*MAT***a**, *tub2-25* (A19K-T23V-G26D-Y270F), *erg6::HIS3, pdr1::hygB, pdr3::NAT, his3Δ200, leu2Δ1, ura3-52, trp1Δ63, pMET3-3xHA-CDC20-TRP1, mad2Δ::KanR, GFP-TUB1-URA3*

All strains except MGY50 (control S288C strain) were created in this study.
